# Effective Automated Feature Construction and Selection for Classification of Biological Sequences

**DOI:** 10.1371/journal.pone.0099982

**Published:** 2014-07-17

**Authors:** Uday Kamath, Kenneth De Jong, Amarda Shehu

**Affiliations:** 1 Computer Science, George Mason University, Fairfax, Virginia, United States of America; 2 Krasnow Institute, George Mason University, Fairfax, Virginia, United States of America; 3 Bioengineering, George Mason University, Fairfax, Virginia, United States of America; 4 School of Systems Biology, George Mason University, Fairfax, Virginia, United States of America; UMR-S665, INSERM, Université Paris Diderot, INTS, France

## Abstract

**Background:**

Many open problems in bioinformatics involve elucidating underlying functional signals in biological sequences. DNA sequences, in particular, are characterized by rich architectures in which functional signals are increasingly found to combine local and distal interactions at the nucleotide level. Problems of interest include detection of regulatory regions, splice sites, exons, hypersensitive sites, and more. These problems naturally lend themselves to formulation as classification problems in machine learning. When classification is based on features extracted from the sequences under investigation, success is critically dependent on the chosen set of features.

**Methodology:**

We present an algorithmic framework (EFFECT) for automated detection of functional signals in biological sequences. We focus here on classification problems involving DNA sequences which state-of-the-art work in machine learning shows to be challenging and involve complex combinations of local and distal features. EFFECT uses a two-stage process to first construct a set of candidate sequence-based features and then select a most effective subset for the classification task at hand. Both stages make heavy use of evolutionary algorithms to efficiently guide the search towards informative features capable of discriminating between sequences that contain a particular functional signal and those that do not.

**Results:**

To demonstrate its generality, EFFECT is applied to three separate problems of importance in DNA research: the recognition of hypersensitive sites, splice sites, and ALU sites. Comparisons with state-of-the-art algorithms show that the framework is both general and powerful. In addition, a detailed analysis of the constructed features shows that they contain valuable biological information about DNA architecture, allowing biologists and other researchers to directly inspect the features and potentially use the insights obtained to assist wet-laboratory studies on retainment or modification of a specific signal. Code, documentation, and all data for the applications presented here are provided for the community at http://www.cs.gmu.edu/~ashehu/?q=OurTools.

## Introduction

The wealth of biological sequences made possible by high-throughput sequencing technologies is in turn increasing the need for computational techniques to automate sequence analysis. In particular, as the community at large is focusing on elucidating the sequence-function relationship in biological macromolecules, a primary sequence analysis problem involves unraveling the rich architecture of DNA and mapping underlying functional components in a DNA sequence [1]. A combination of valuable biological insight gathered from wet-laboratory experiments and increasingly powerful computational tools has resulted in significant progress being made in important sequence analysis tasks, such as gene finding [2], [3]. Despite this progress, challenges remain [4], [5]. For instance, accuracy in gene finding ultimately depends on addressing various subproblems, one of which is the correct detection of splice sites that mark the beginning and end of a gene. The splice site prediction problem is now considered a primary subtask in gene finding and is thus the subject of many machine learning methods [6]–[16]. Other prominent DNA analysis problems involve the identification of regulatory regions [17], [18] through detection of binding sites of transcription factors [19]–[21] or detection of hypersensitive sites as reliable markers of regulatory regions [15], [22]–[28], identification of ALU sites [29]–[33] to understand human evolution and inherited disease [34], [35], and more.

From a computational point of view, detecting specific functional regions in a DNA sequence poses the interesting and challenging task of searching for signals hidden in sequence data. Detecting a signal in a given sequence or whether a sequence contains a particular signal is a difficult computational task, particularly in the *ab initio* setting, for which little or no *a priori* information is available on what local or distal interactions among the building blocks of investigated sequences constitute the sought signal. Yet, automating this process is central to our quest to understand the biology of organisms and characterize the role of macromolecules in the inner workings of a healthy and diseased cell. This quest is not limited to nucleic acids. Important sequence analysis problems include predicting protein solubility, crystallizability, subcellular localization, detecting enzymatic activity, antimicrobial activity, secondary structure folding, and more [36]–[46]. The focus in this paper on DNA is due to a growing body of work in machine learning pointing to the fact that many important functional signals consist of a complex combination of local and distal information at the nuucleotide level.

Sequence analysis problems in which the objective is to find what constitutes a functional signal or property at the sequence level naturally lend themselves to formulation as classification problems in machine learning. The effectiveness of these algorithms largely depends on the feature sets used. In some settings, the construction of effective features can be facilitated by *a priori* insight from biologists or other domain experts. For instance, biophysical insights have been instrumental in developing effective features for predicting protein subcellular localization and folding rates, CG islands in DNA sequences, and more [37], [38], [42], [43], [47].

However, it is becoming increasingly clear that there are problems for which domain-specific insight is either incomplete or hard to translate into effective features. As a consequence, there is considerable interest in automating the process of constructing effective features. A prominent example is the automated detection of splice sites in DNA sequences [12]–[16]. The key issue here is how to define a space of potential features that is sufficiently rich to allow the generation of effective features while maintaining computational feasibility.

In recent work, we have indicated how one can explore large spaces of potential features in a computationally-viable manner by employing evolutionary algorithms (EAs) [15], [16]. The success of this "proof of principle" effort has prompted us to propose and investigate a more general EA-based framework (EFFECT) for efficient automated feature construction for classification of biological sequences. In this paper we describe the generalizations and then demonstrate the broad applicability of the framework on three DNA sequences analysis problems on the detection of splice sites, HS sites, and ALU sites in DNA sequences. The algorithmic realizations of EFFECT for each of the selected problems in this paper are sufficiently detailed to allow one to adapt the framework for other sequence classification problems of interest. Indeed, one of the contributions of this work is in providing a roadmap as to how one can do so in different application settings. To further facilitate this, the entire data, code, and documentation are provided to the community at http://www.cs.gmu.edu/~ashehu/?q=OurTools.

The rest of this article is organized as follows. We first provide a brief review of related research that includes machine learning methods for classification of biological sequences and EAs for feature construction in the context of classification. The EFFECT framework is detailed in Methodology. A comprehensive analysis of results from application of this framework on the three chosen problems is presented in Results. The paper concludes in Discussion, where we provide a short summary of the main features of EFFECT, its availability to the research community, and its use for other classification problem of interest.

### Related Work

#### Methods for Classification of Sequence Data

We focus here on supervised learning methods for classification of sequences. In this scenario, a model is trained to find features that separate *labeled training* sequence data. Typically, these are binary classification problems in which a *positive* label is assigned to sequences known to contain a particular functional signal or property, and a *negative* label to sequences that do not. The learned model is then applied to novel sequences to make label predictions and thus *detect* or *recognize* the presence of the sought functional signal.

Our review below categorizes classification methods into statistical-based and feature-based, though many methods are a combination of the two approaches. Typically, the process involves first transforming sequence data into vectors over which an underlying classifier operates. In statistical-based approaches, the focus is on the underlying statistical model for the classification. In feature-based approaches, the primary focus is on constructing effective features that allow transforming sequence data into (feature) vectors for standard classifiers. What follows below is not a comprehensive review of literature on each of these two approaches, but rather a summary of representative methods in each category to facilitate the discussion of results in the comparison of our framework to state-of-the-art methods.

#### Statistical Learning Methods

Statistical learning methods can be broadly classified by the models that they employ, which can be generative or discriminative. Generative models learn the joint probability 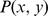 of inputs 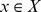 with labels 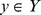. Bayes rule is used to then calculate the posterior 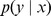 and predict the most likely label for an unlabeled input. Discriminative models learn the posterior directly, but this also limits them to a supervised setting that demands labeled training data (as opposed to the ability of generative models to additionally exploit unlabeled data). Nonetheless, discriminative models are preferred in many classification settings, as they provide a more direct way at modeling the posterior without first addressing a more general setting (as demanded by modeling the joint probability) [48]. The transformation of input sequence data into numeric data for these models is conducted *a priori* through a kernel function or a feature-based method explicitly extracting features of relevance for the transformation.

Heuristic procedures have been proposed to combine discriminative and generative models [49] as a way to address the issue that generative methods lose their ability to exploit unlabeled data when trained discriminatively [50]. The resulting hybrid methods have been shown to result in superior performance on recognition of transcription factor-binding sites on DNA [51]. Representative methods include the position-specific scoring matrix (PSSM) – also known as the position-weight matrix (PWM) - a method that assumes nucleotides at all positions are drawn independently [52], [53], the weight array model (WAM) which relaxes assumptions of independence by additionally modeling dependencies on a previous position [54], higher-order Markov models which model more dependencies and outperform PSSMs [55], [56], and even more complex models like Bayesian networks [57], [58] and Markov Random Fields (MRFs) [59], [60]. A mixture of Bayesian trees and PSSMs in [61], smooth interpolations of PSSMs, and empirical distributions [62] have also been proposed to model arbitrary dependencies.

#### Kernel-based Methods

SVMs are probably the most widespread discriminative learning method in bioinformatics due to their ease of implementation and solid grounding in statistical theory [63], [64]. They have been applied to many sequence classification problems, including prediction of transcription start sites on DNA [65], translation initiation sites [66], gene finding [67], transcription factor-binding sites [68], and DNA regulatory regions [69]. The predictive power of SVMs greatly depends on the chosen kernel function. This function maps input (sequence, here) data onto a usually higher-dimensional feature space where provided samples of the two classes can be linearly separated by a hyper-plane. Many kernels are designed for sequence classification, of which the most relevant and state-of-the-art are weighted position and weighted position with shift kernels devised for recognition of DNA splice sites [12]. In these kernels, limited-range dependencies between neighboring nucleotides are considered to encode features for the SVM. Concepts from evolutionary computation have been lately proposed to learn effective, possibly more complex, kernels for a particular sequence classification problem at hand [27], [28].

#### Feature-based Methods

Feature-based methods make the process of feature construction transparent and so can offer constructed features for inspection and further analysis to biologists. Constructing effective features, however, is non-trivial. The straightforward approach is to use enumeration to list all considered features. When no domain-specific expertise is available to guide feature construction towards certain feature types, the predominant approach has been to limit the focus to features that are strings of 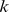 symbols over the alphabet of building blocks in considered biological sequences (nucleotides in DNA/RNA and amino acids in proteins). These k-mers are also known as spectrum features [70].

The essential idea is to transform given sequences into numeric vectors recording frequency or occurrence of k-mers and then employ supervised learning techniques, such as SVMs, to separate training data in the resulting vector space [25]. Spectrum features have been shown useful in various classification problems, such as prediction of DNA promoter regions, cis sites, HS sites, splice sites, and more [70]–[73]. However, work has shown that the majority of spectrum features are seldom useful and can be removed by effective feature selection algorithms [74].

In many classification problems on biological sequences, research has shown that simple spectrum (compositional-based) features are not sufficient. Problems, such as predicting protein enzymatic activity, DNA hypersensitive sites, or RNA/DNA splice sites seem to necessitate complex local and distal features [11], [13], [14], [25]–[28], [38]. In particular, taking into account dependencies through features that encode correlations or simultaneous occurrences of particular 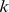-mers at different positions in a biological sequence is shown to be important for accurate detection of splice sites [14]–[16]. Work in [8], [14] introduced the idea of explicitly considering various feature types in the context of splice site detection but limited the number of types and number of enumerated features per type to control the size of the feature space and the computational cost demanded by enumeration. The feature types considered were position-based, region-based, and composition-based [14].

In general, enumeration-based approaches introduce artificial limits on the length and the complexity of features in order to achieve reasonable computation times. Moreover, insight in a particular problem domain is difficult to translate into meaningful features when a combination of local and distal features are needed. Ideally, a general feature construction approach would be able to operate *ab initio*; that is, explore the space of possible local and distal features and guide itself towards discriminating features. When the types or number of features are not limited, one is invariably confronted with a feature construction problem that is NP-hard problem due to the combinatorial explosion in the size of the feature space [75]. Yet, a variety of general purpose search techniques have been shown effective for NP-hard problems. In particular, EAs, which we summarize next, provide a viable alternative for exploration of complex feature spaces in automated feature construction and are the backbone of the framework proposed here for automatic feature construction for classification of biological sequences.

### EAs for Exploration of Feature Spaces

The ability of EAs to efficiently explore large search spaces with complex fitness landscapes makes them appealing for feature construction [76]. EAs mimic biological evolution in their search for solutions to a given optimization problem. Typically, a population of candidate solutions, also referred to as individuals, is evolved towards the true ones through a process that generates candidate solutions and retains only a population deemed promising according to some *fitness* function.

Recognized early for their promise in addressing difficult optimization problems [77], EAs have gained popularity for feature construction in different application settings [16], [28], [38], [78]–[83]. In particular, recent work has shown improved classification accuracies when using genetic algorithms (GAs), a class of EAs, to replace feature enumeration techniques in predicting promoter regions, HS sites, and splice sites in DNA, and even enzymatic activity in proteins [15], [16], [26]–[28], [38].

In standard GAs, individuals are fixed-length strings of symbols. In another class of EAs, genetic programming (GP) algorithms, an individual is a variable-length tree composed of functions and variables. The functions are represented as non-terminal nodes, and the variables represented as terminal (leaf) nodes. GPs were originally introduced to evolve computer programs and complex functions [84]–[87]. Today, GP-based algorithms are being used for a variety of applications, including feature construction in the context of classification of biological sequences [38], [88]–[92]. Our recent work introduced a GP-based method for feature construction in the context of DNA splice site recognition [16]. In this paper, we present a more general EA-based approach that makes use of a GP algorithm to explore complex feature spaces and generate predictive features from sequence data.

### Methods for Feature Selection

EAs can be used to construct a large set of discriminating features, but selecting a non-redundant subset that retains its predictive power remains a difficult and open problem, particularly when the set of features is large [93]. Finding an optimal set of features is generally intractable [94] and is shown to be NP-hard in various settings [95], [96]. This is due in part to the fact that a feature by itself may not be predictive of a particular class but may be informative in combination with other features. Additionally, features which are informative by themselves may be redundant when grouped with others. In general, even finding a small subset of discriminating features is a challenging search problem [93], [97], [98].

Feature selection methods generally follow one of two approaches, subset search and subset evaluation [99]. Univariate feature selection like Information Gain or Chi-square are not very useful when applied on a set of features that already have high discriminatory power, as is the case with features found by the GP algorithm employed in the first stage of our EFFECT framework. In cases of already discriminating features, a more relevant criterion for feature selection is to reduce redundancy while retaining predictive power in the selected subset. In this paper we present an EA-based approach to feature selection that achieves that goal.

## Methodology

**Figure 1:**
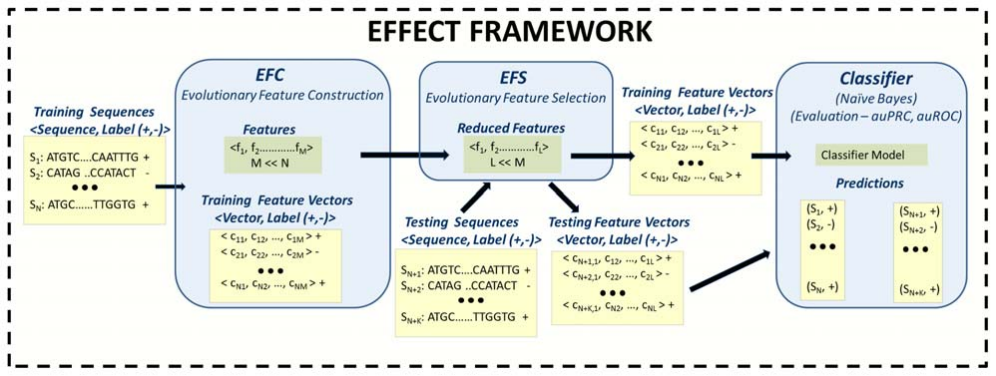
The EFFECT framework consists of two algorithms, EFC and EFS, as detailed in the Methods section. While EFC conducts a biased exploration of a vast space of potentially complex features to find a set of top features, EFS reduces this set to a subset of informative yet low redundancy features. The remaining features are used to transform sequence data into vector data that can be separated by any classifier.

The proposed EFFECT framework consists of two stages, each comprised of an EA. In the first stage, the Evolutionary Feature Construction (EFC) algorithm is used to search a given space of complex features and identify a set of features estimated to be effective in the context of a given classification problem. These features are then fed to the second stage, where a second algorithm, Evolutionary Feature Selection (EFS), reduces the set of constructed features by selecting a subset deemed most informative without sacrificing performance. A schematic of the framework showing the interplay between these two algorithms, is shown in Figure 1.

### Constructing Complex Features with EFC

Since EFC is a generalization of the feature generation algorithm presented in [16], our description here focuses primarily on the novel components, providing a brief summary of the common elements where needed and directing the reader to Text S1 for further details.

Central to the power of EFC is its generalized representation of sequence-based features as GP trees. These feature "trees" are maintained in a population that evolves over generations using standard GP reproductive mechanisms of mutation and crossover. Mimicking the process of natural selection, features that are deemed more discriminative for classification have a higher probability of surviving into the next generation, steering the probabilistic search in EFC towards more effective features. The discriminative power of a feature is estimated through an empirical or *surrogate* fitness function. The best features (those with highest fitness) found by EFC are collected in a set referred to as a hall of fame. It is this set that is fed to the subsequent EFS algorithm for feature subset selection.

#### Feature Representation in EFC

As standard in GP, the individuals (features) evolved by the EFC algorithm are represented as parse trees [87]. In EFC, the leaf nodes of a **feature tree** are known building blocks of given biological sequences. In the case of DNA sequences, for instance, these blocks are the four nucleotides in the DNA alphabet. To improve on generality and effectiveness, EFC supports additional building blocks that represent *groups of nucleotides* based on similar chemical properties. In this paper, this capability is illustrated by the use of the IUPAC code [100], resulting in 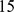 symbols listed in Table 1. If the sequences of interest are proteins, the building blocks can either be amino-acid identities, types, or other categorizations based on physico-chemical properties.

**Table 1:** IUPAC code is adapted from [100].

Symbol	Meaning	Description Origin
G	G	**G**uanine
A	A	**A**denine
T	T	**Thymine**
C	C	**C**ytosine
R	G or A	pu**R**ine
Y	T or C	p**Y**rimidine
M	A or C	a**M**ino
K	G or T	**K**etone
S	G or C	**S**trong interaction
W	A or T	**W**eak interaction
H	A or C or T	**H** follows G in alphabet
B	G or T or C	**B** follows A in alphabet
V	G or C or A	**V** follows U in alphabet
D	G or A or T	**D** follows C in alphabet
N	G or A or T or C	a**N**y

Alternatively, building blocks can be short subsequences or **motifs** of 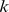 symbols. Information may be available from domain experts to determine the length of these motifs. For instance, work in splice sites shows that motifs of length 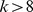 are not useful [13], [14]. In other applications, there may be lower bounds on the length of effective motifs. Such bounds may be available and specified *a priori* to EFC or tuned interactively after analysis of constructed features. In the selected applications of the EFFECT framework in this paper, the leaf nodes of feature trees are motifs, and we limited the length of these motifs between 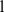 and 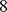.

**Figure 2:**
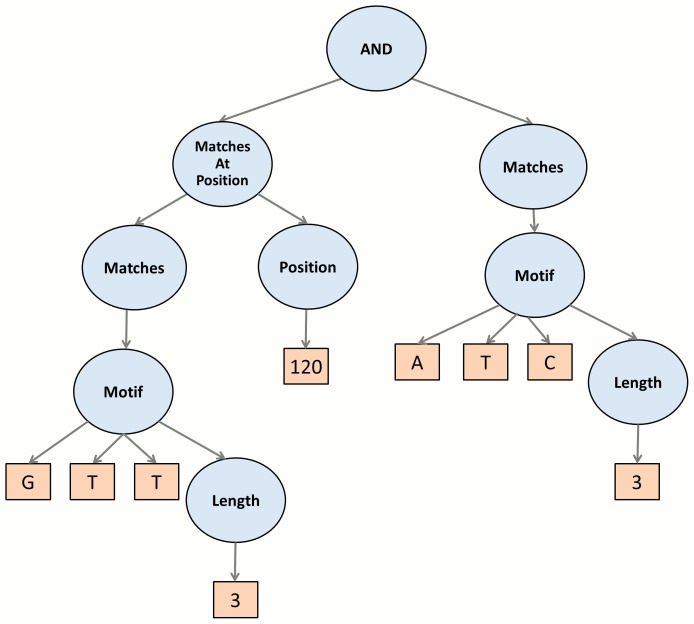
Conjunction Features combining one positional and one compositional feature.

**Figure 3:**
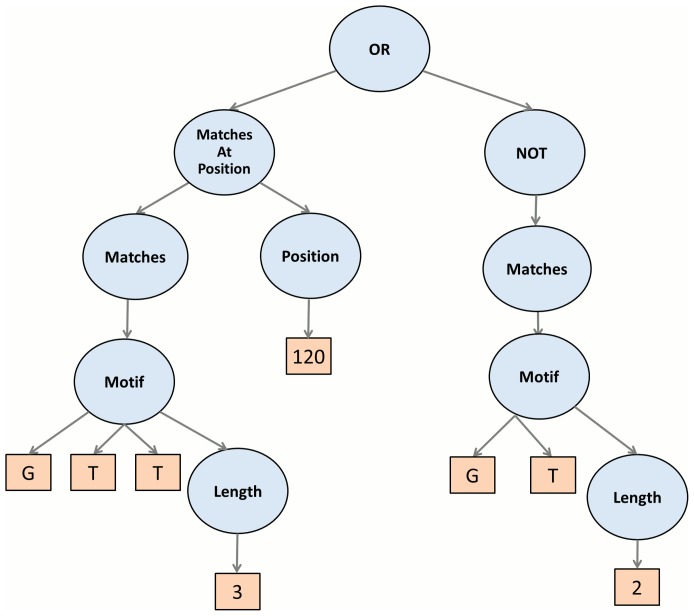
Disjunction Features combining one positional and one negation of compositional feature.

As illustrated in Figure 2 and Figure 3. EFC uses the standard boolean operators (and, or, not) to combine basic building blocks into more complex features. In addition to boolean operators, the EFC algorithm uses application-specific functional nodes to assist in the construct meaningful features for biological sequences. These are listed in Table 2. An important *functional generalization* in EFC is the ability to specify the matching of a motif in some region (up or down) or matching it around some expected position. This allows for the construction of features that are more robust to possible sequence variations. In Text S1 we provide more detail regarding the types of features that one can construct with these operators and provide illustrations for them.

**Table 2:** A table of non-terminals and terminals employed in feature construction.

Name	Args	Return Type
AND	2 non-terminal boolean	Boolean
OR	2 non-terminal boolean	Boolean
NOT	2 non-terminal boolean	Boolean
Correlational	2 non-terminal boolean, Shift	Boolean
Matches	Motif	Boolean
MatchesAtPosition	Matches, Position	Boolean
MatchesAtPositionWithShift	Motif, Position,Shift	Boolean
MatchesAtRegion	Matches, Region	Boolean
Motif-*	ERC-chars	Motif
Shift	ERC-int	Integer
Region	ERC-int	Integer
Length	ERC-int	Integer
ERC-char	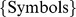	Character
ERC-int	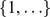	Integer

#### Population and Generation Mechanism

As detailed in Text S1, the initial population of 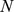 features is carefully constructed to contain a variety of tree shapes with maximum depth 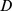. In contrast to EAs with fixed population sizes, EFC employs an implosion mechanism that reduces the size of the population by 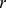
% over the previous one, in order to avoid known convergence pitfalls of GPs. The population of features evolves for a pre-specified number of generations 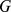. Each population contributes its top 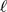 features to a *hall of fame*. In turn, the hall of fame is used to provide a randomly selected initial set of 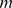 features for the next generation, with the rest of the features in the next generation obtained through reproductive operators.

In the experiments reported in this paper, 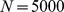, 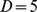, 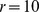, 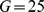, 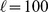, and 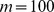.

#### Reproductive Operators

Based on studies that show robust EAs incorporate both asexual (mutation) and sexual (crossover) breeding operators [101], EFC employs both operators. These operators are executed until the goal population size for the next generation is reached. Each of the operators has a certain probability with which it is performed. Given the additional functional nodes in EFC over our prior work in [16], four new mutation operators are employed depending on the type of tree node being modified. Each of the variants has equal probability of being performed once the mutation operator is selected. Additional details and illustrations on the mutation and crossover operators are provided in Text S1.

#### Bloat Control

A common problem with tree-based individuals in EAs is that, as generations progress, individuals become more complex without any improvement in fitness. This is known as *bloat*. It is important to control bloat, particularly when the goal is to have features that are easily interpretable by humans. As such, bloat control is an important element in EFC, the details of which are given in Text S1.

#### Fitness Function

EFC employs a surrogate fitness function or a "filter" approach, which is considered to be more effective than wrapper approaches for feature evaluation [102]. Since most sequence classification datasets are imbalanced in the sense of having very few positives as compared to a large number of negatives, the objective of a filter approach is to improve precision while managing the discriminative power of features. For this purpose, we use the following fitness function: 
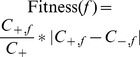
. In this equation, 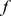 refers to a particular feature, 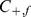 and 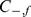 are the number of positive and negative training sequences that contain feature 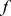, respectively, and 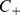 is the total number of positive training sequences. This fitness function tracks the occurrence of a feature in positive sequences, as negative sequences may not have any common features or signals. The fitness function additionally penalizes non-discriminating features; that is, features that are equally found in positive and negative training sequences.

#### Hall of Fame

Previous research on EAs has noted that if parents die after producing offspring, there can be *genetic drift* or convergence to some local optimum [76]. This can result in the loss of some of the best individuals. The EFC algorithm addresses this issue by using an external storage of features known as a *hall of fame*. As noted above, the 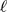 best individuals in every generation are added to the hall of fame, and the hall of fame in return helps seed the population in each generation with 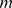 randomly selected features. It should be noted that the parameter values for 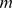 and 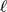 should depend on the problem at hand. In general, keeping the fittest individuals in a hall of fame improves overall performance [103]. After execution of the EFC, the features in the hall of fame are those submitted to the ensuing EFS algorithm.

### Effective Feature Selection with EFS

The hall of fame features generated by EFC were selected on the basis of their individual performance. What is required for effective and efficient classification is to identify a relevant and non-redundant subset of features. EFS, a novel GA-based algorithm, is employed for this purpose and described below.

#### Feature Subset Representation in EFS

EFS evolves feature subsets by having individuals in the population correspond to feature subsets represented as binary strings. The length of each string is equal to the number of individuals in the hall of fame. A string of all '1's would correspond to the maximum subset, the hall of fame itself, and a string of all '0's would correspond to the empty subset. In addition to being a suitable representation for our purposes, binary representations in GAs are the standard ones and include a well-studied set of mutation and crossover operators.

#### Population and Generation Mechanism

The initial population contains 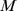 individuals of length 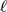 which are created using randomly generated binary strings and represent 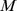 subsets of selected features from the hall of fame. The GA implementation in EFS is generational; that is, after the offsprings are created using mutation and crossover, the parents die. The population size of 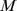 remains constant throughout the generations in EFS. The number of generations is set to 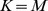 by default. The best individual (feature subset) is tracked over the generations and constitutes the feature subset presented to a classifier for labeling new unlabeled (testing) sequences. For the experiments reported in this paper, 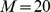.

#### Reproductive Operators

EFS uses a standard bit-flip mutation operator with mutation rate of 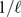. Additionally, standard uniform crossover is used, in which each bit is considered a crossover point with a probability of 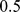. It has been shown that employing uniform crossover along with bit-flip mutation is effective at balancing exploration and exploitation of search landscapes [101]. Parent(s) for the reproductive operators are selected using standard fitness-proportional selection. Details are provided in Text S1.

#### Fitness Function

Recall that the objective of EFS is to find a subset of features with high feature-class correlation to retain discriminating power but low feature-feature correlation to reduce redundancy. EFS achieves this by employing a correlation-based fitness function [104]. Using a measure of feature correlation 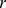 based on Pearsons correlation, a set of features 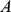, a feature subset 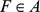, and a to-be-predicted class 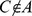, let the average feature-class correlation be
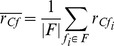
(1)


Feature-feature correlation is given by
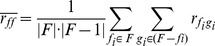
(2)


Combining the two for maximizing class-feature correlation while minimizing feature-feature correlation and weighing with the number of features 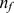, results in the following fitness function:
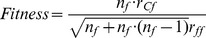
(3)


### Classifiers

The best (highest fitness) individual obtained from EFS defines the feature subset to be used by a machine learning classifier. Generally, any classifier can be used, and our experimentation shows there are no significant differences among standard ones. Since the Naive Bayes (NB) classifier is the simplest, fastest, and most effective when features have low correlation among them but high correlation with class [105], [106], we employ NB as our classifier of choice. We used the kernel density estimator with NB using Weka, which is the default estimation method.

### Experimental Setting, Implementation Details, and Performance Measurements for Analysis

#### Experimental Setting

The experimental setting has been designed to support two forms of analysis. First, the features generated by EFFECT are made available for visual inspection and detailed analysis. Second, the experimental setting allows for a detailed analysis of the classification performance of a Naive Bayes classifier using EFFECT-generated features in comparison to a representative set of alternative machine learning approaches (as described in the Related Work section). First, a baseline feature-based method is defined that uses spectrum (compositional) features and over-represented motifs as reported from alignments using Gibbs sampling. The features are fed to the same Naive Bayes classifier used to evaluate EFFECT-obtained features for a direct comparison of features in the context of classification. A comprehensive comparison is also conducted with state-of-the-art statistical methods, PSSM, WAM, Bayes Tree Network with PWM, Markov Chain (MC), and Maximum Supervised Posterior (MSP). Their implementation is made possible through the Jstacs software package [107]. MSP is configured with PWM and Homogenous HMM classifiers as generative mixture classifier. EFFECT is also compared to kernel methods. We focus on the latest two most successful kernel methods (shown so on the splice site prediction problem [12]), the weighted degree positional kernel (WD) and the weighted degree positional kernel with shift (WDS) method (the underlying classifier is an SVM).

To the extent possible, the methods selected for comparison have been tuned in order to obtain their best performance on each of the data sets considered in this paper, often in communication with the original developers. Details on our tuning protocols and resulting parameter values are posted on the http://www.cs.gmu.edu/~ashehu/?q=OurTools site we provide that lists the EFFECT code, documentation, and data sets.

#### Implementation Details

All experiments are performed on an INTEL 2X 4core machine with 3.2 Ghz and 8GB of RAM. The code for the EFC algorithm in EFFECT is written in Java, using the publicly-available ECJ toolkit [108] and BioJava [109] software packages. The code for the EFS algorithm in EFFECT is also in Java, using the GeneticSearch and CFSSubset techniques of the publicly-available WEKA package for machine learning. The implementation of the statistical methods employed for comparison is in Java, based on the publicly-available Jstacs package [107]. The kernel-based methods are implemented using the publicly-available Shogun toolkit [110] with the standard SVM implementation provided in the publicly-available LibSVM package [111]. The feature-based methods employed for a baseline validation are implemented in Java. The resulting open source software that we provide to the community for academic purpose includes not only the EFFECT framework, but also our implementations of all the methods employed for comparison, tuned parameters, along with datasets, features, and complete models.

#### Performance Measurements

Standard datasets used by other researchers are used in each of the three application settings showing the generality and power of the EFFECT framework. Since most of these datasets have an imbalance between the size of the positive and negative classes, classification accuracy is a meaningless performance measurement. For this reason, the analysis in this paper employs other evaluation criteria, such as area under the Receiver Operating Characteristic Curve (auROC) and area under the Precision Recall Curve (auPRC). All these are based on the basic notions of TP, FP, TN, and FN, which correspond to number of true positives, false positives, true negatives, and false negatives. Details on common performance measurements for classification can be found in [112]. To briefly summarize what these measures capture, consider that predicted instances (sequences assigned a label by the classification model) can be ordered from most to least confident. Given a particular confidence threshold, the data above the threshold can be considered correctly labeled. The true positive rate and false negative rate can then be computed as one varies this threshold from 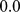 to 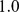. In an ROC, one typically plots the true positive rate (TPR = TP/(TP+FN)) as a function of the false negative rate (FNR = FN/(FN +TN)). The auROC is a summary measure that indicates whether prediction performance is close to random (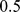) or perfect (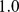). Further details can be found in [112].

For unbalanced datasets, the auROC can be a wrong indicator of prediction, since this measure is independent of class size ratios; large auROC values may not necessarily indicate good performance. The auPRC is a better measure for performance when the class distribution is heavily unbalanced [113]. The PRC measures the fraction of negatives misclassified as positives and so plots the precision (TP/(TP+FP)) vs. the recall ratio (this is TPR, sometimes referred to as sensitivity). Again, as one varies the threshold, precision can be calculated at the threshold that achieves that recall ratio. auPRC is a less forgiving measure, and a high value indicates that a classification model makes very few mistakes. Thus, the higher the auPRC value, the better.

Performance is measured and compared to all methods used for comparison both on training and testing datasets. When testing datasets are not available for a particular application, 10-fold cross-validation is conducted instead. We used 1% of training data with equal mix of both classes as the evaluation set for tuning every employed for comparison, reserving the rest 99% of training data for cross-validation. The idea is to train on a randomly-selected 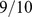
ths of the data and then test on the rest. This is repeated 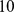 times, and an average performance is reported in terms of the evaluation criteria described above. Moreover, since the EFFECT framework employs stochastic search algorithms (EFC and EFS), it is run 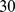 times, thus resulting in 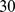 sets of features. Each set is evaluated in the context of classification performance (using NB). The reported performance measurements are averages over 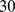 values obtained (over each set of features from a run of EFFECT). Paired t-tests are used to measure statistical significance at 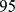
% confidence intervals.

It should be noted that many of the statistical learning (and kernel) methods used for comparison in this paper have a limitation of demanding that all input sequences be of fixed length. On the other hand, some of the datasets available consist of sequences of variable length. Typically, in such a setting, one can either use a random alphabet to “fill” smaller sequences and achieve a maximum fixed length or throw away shorter sequences. Since shorter sequences make up only 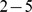
% of the datasets under each application in this paper, we decide to discard shorter sequences (additionally, our analysis indicates that doing so results in better performance than filling sequences with random alphabets). We also point out that the parameters of each of the methods used for comparison have been tuned to achieve a maximum performance for each method. Various classifier parameters (e.g., the cost parameter C in SVM) have also been tuned for this purpose. All tuned parameters are listed at http://www.cs.gmu.edu/~ashehu/?q=OurTools.

## Results

We summarize the performance of EFFECT on each of the three selected applications on DNA sequence analysis, recognition of HS, splice, and ALU sites. The training (and testing, where available) datasets employed are detailed first, followed by an empirical analysis of the results on each application.

### Datasets

Benchmark data sets are selected for each of the three application settings in order to allow comparison with as many methods as possible.

#### Datasets for Recognition of HS sites

The dataset employed for evaluating the features constructed in EFC and for training the NB classifier is the one provided at noble.gs.washington.edu/proj/hs. This dataset consists of experimentally-determined sequences (each 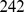 nucleotides long) extracted from the human genome and consists of 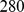 HS and 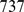 non-HS ones. THe HS sequences were identified employing cloning and in-vivo activity of K562 erythroid cells [114], whereas the non-HS sequences were sequences collected and distributed proportionally throughout the human genome but found not to be hypersensitive when tested in the same cell type.

#### Datasets for Recognition of Splice Sites

A distinction is made between acceptor and donor splices sites. An acceptor splice site marks the start of an exon, whereas a donor splice site marks the end. These sites have different consensus sequences, and machine learning research has additionally shown they have different features of relevance for classification [13], [16]. For the purpose of feature construction and classification performance, splice site datasets are split into a donor subset and an acceptor subset, and evaluation is done separately on each subset.

The splice site recognition problem is well-studied in machine learning, and so many datasets have been accumulated over the years. We report performance on three datasets used as benchmarks in recent literature. The first dataset is known as NN269 to indicate that it is extracted from 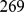 human genes [115]. It consists of 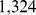 confirmed acceptor sequences, 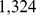 confirmed donor sequences, 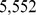 false acceptor sequences, and 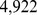 false sequences (length of acceptor sequences is 90 nucleotides, whereas that of donor sequences is 15 nucleotides). Further details on these sequences can be found in [115]. We split this dataset into a training and testing dataset. The training dataset has 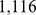 true acceptor, 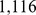 true donor, 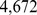 false acceptor, and 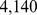 false donor sequences. The testing dataset has 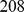 true acceptor, 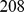 true donor, 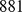 false acceptor, and 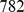 false donor sequences.

Performance is reported on another dataset extracted from the C_Elegans (worm) genome, prepared as in [12], on which statistically-significant differences are observed in the comparative analysis between EFFECT and other methods. Briefly, the genome is aligned through blat with all known cDNA sequences available at http://www.wormbase.org and all known EST sequences in [116] to reveal splicing sites. 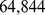 donor and 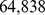 acceptor sequences, each 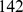 nucleotides long are then extracted from the alignment, centered at the identified splicing sites. Equal-length negative training sequences are centered around non-splice sites (selected in intronic regions). In [12], 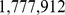 negative acceptor and 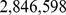 negative donor sequences are constructed. This dataset is too big to feasibly conduct a thorough comparative analysis with other methods and gather summary statistics over many runs. For this reason, we sample a smaller training set of 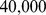 sequences from the entire (positive and negative) dataset, preserving the ratio of positive to negative sequences as in the original dataset.

#### Datasets for Recognition of ALU Sites

319 known ALU sequences were obtained from NCBI website. This small set of sequences is considered to be representative of 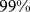 of all ALU sequences in GenBank [117]. The average length is approximately 300 nucleotides. A negative training dataset of 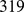 sequences was constructed at random, sampling similar-length sequences with similar nucleotide distribution as that found over ALU sequences.

### Comparative Analysis

#### Empirical Analysis on Recognition of HS Sites

Given the availability of only a training set in this setting, 10-fold validation is used to measure the classification performance of the NB classifier on features obtained through EFFECT and compare it to the other methods summarized above. We recall that EFFECT is run 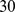 independent times, and the auROC and auPRC measurements reported are averages over these runs, as well. Table 3 compares EFFECT to all the methods employed for comparison in terms of auROC and auPRC values. As Table 3 shows, EFFECT achieves the highest performance both in terms of auROC (89.7%) and auPRC (89.2%). For comparison, MSP achieves the second highest auROC (85.5%), and K-mer (feature-based with spectrum features in SVM) achieves the second highest auPRC (82.6%). Paired t-tests at 95% confidence intervals indicate that the reported values for EFFECT are statistically significant (data not shown). Taken together, this comparative analysis demonstrates that the quality of the features found by EFFECT is such that even a simple classifier, such as NB, achieves comparable classification performance with sophisticated methods for HSS recognition.

**Table 3:** auROC and auPRC comparison analysis for Recognition of HSS Sites.

Algorithm	auROC	auPRC
*Feature-based*		
K-mer	82.20	82.6
Gibbs Sampling	79.3	50.3
EFFECT	**89.7**	**89.2**
*Statistical-based*		
PWM-HMM	70.8	47.8
BayesNetwork	72.5	49.5
HomogenousHMM	82.02	71.5
WAM-HMM	80.05	70.0
MSP	85.5	72.9
*Kernel-based*		
WeightedPosition	80.01	62.3
WeightedPositionShift	80.93	64.9

#### Empirical Analysis on Recognition of Splice Sites

Our analysis first proceeds on the NN269 dataset. We recall that the analysis (as well as construction and selection of features and training of classifiers) is conducted separately for the acceptor and donor datasets. Table 4 compares auROC and auPRC values obtained on the testing sequences in each dataset. While EFFECT and the kernel-based methods have the highest performance (EFFECT is second best) in both auROC and auPRC on the acceptor dataset, and all methods are comparable on the donor dataset (with the exception of inhomogeneous HMM and K-mer, which perform worst), the t-test analysis indicates none of the methods' performance is statistically significant on the NN269 splice site dataset.

**Table 4:** auROC and auPRC comparison analysis for recognition of splice sites on NN269 dataset.

	*ACCEPTOR*		*DONOR*	
Algorithm	auROC	auPRC	auROC	AuPRC
*Feature*				
K-mer	63.3	75.5	90.8	90.1
Gibbs Sampling	62.8	72.4	88.8	90.5
EFFECT	97.7	94.3	98.2	92.81
*Statistical*				
PWM	97.1	90.6	97.7	91.9
BayesNetwork	97.25	90.6	97.7	90.9
HomogenousHMM	59.2	26.3	86.3	71.5
InHomogenousHMM	96.78	88.41	98.18	92.42
MSP				
*Kernel*				
WeightedDegreePosition	98.16	92.53	98.5	92.86
WeightedDegreePositionShift	98.65	94.36	98.13	92.47

In a second analysis, the C_Elegans splice site dataset is employed as a training dataset. 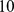-fold validation on highly unbalanced positive over negative datasets (the positive dataset in both the donor and acceptor setting is about 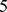
% of the entire dataset) clearly separates performance among the different methods. Table 5 shows that EFFECT and kernel-based methods achieve the highest performance in terms of auROC on the acceptor dataset (around 99% for kernel-based and 98% for EFFECT). The two are top performers in terms of auROC on the donor dataset, as well (close to 100% for kernel-based and 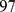
% for EFFECT). However, the unbalancing of the positive and negative datasets in each setting results in EFFECT obtaining a higher auPRC value on both the acceptor and donor dataset. On the acceptor dataset, EFFECT obtains an auPRC of 90.2%, followed by kernel-based methods with a value of 89.1% (6 of the 9 methods used for comparison obtain auPRCs less than 16%). On the donor dataset, EFFECT obtains an auPRC of 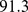
%, followed by kernel-based methods with a value of 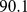
% (5 of the 9 methods used for comparison obtain auPRCs less than 14%). The robust performance of EFFECT even on a highly unbalanced dataset suggests that the bias introduced in the fitness function in the EFC algorithm to improve precision while managing the discriminative power of features gives the algorithm an edge in terms of auPRC.

**Table 5:** auROC and auPRC comparison analysis for recognition of splice sites on *C. elegans* dataset.

	*ACCEPTOR*		*DONOR*	
Algorithm	auROC	auPRC	auROC	auPRC
*Feature*				
K-mer	88.2	15.8	83.1	6.2
Gibbs Sampling	84.2	80.4	79.1	80.3
EFFECT	97.9	**90.2**	96.7	**91.3**
*Statistical*				
PWM	63.6	7.02	62.5	4.8
BayesNetwork	64.2	6.9		
HomogenousHMM	75.03	12.62	78.3	13.9
InHomogenousHMM	75.71	11.3	77.9	12.3
MSP	76.8	13.9	78.21	13.5
*Kernel*				
WeightedDegreePosition	**99.36**	86.7	99.5	88.2
WeightedDegreePositionShift	99.2	89.1	**99.8**	90.1

#### Empirical Analysis on Recognition of ALU Sites

As in the HSS setting, the availability of only a training dataset for the ALU recognition problem limits us to a 10-fold validation. As above, the comparative analysis is conducted only in terms of auROCs, as the ALU training dataset has balanced positive and negative subsets. Table 6 shows that EFFECT achieves the highest performance over the other methods with a mean auROC of 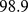
%. For comparison, the second-best value is obtained by one of the kernel-based methods (auROC of 97.8%). Again, values reported by EFFECT are statistically significant, as indicated by a t-test at a 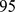
% confidence interval. It is worth noting, additionally, that in the strict context of feature-based methods, EFFECT does not risk overfitting for the NB classifier. Recall that the dataset for ALU sites is small, consisting of 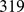 sequences. The number of features should not exceed the size of the dataset. Yet, the number of spectrum features used by a k-mer-based method (with SVM) is 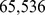, and limiting the number of features with Gibbs sampling still results in 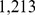 motifs as features.

**Table 6:** auROC and auPRC comparison analysis for Recognition of ALU Sites.

Algorithm	auROC
*Feature*	
K-mer	94.20
Gibbs Sampling	95.2
EFFECT	98.9
*Statistical*	
PWM-HMM	77.45
BayesNetwork	86.82
HomogenousHMM	93.6
WAM-HMM	94.59
MSP	93.54
*Kernel*	
WeightedPosition	96.9
WeightedPositionShift	97.8

### Detailed Analysis of Features Obtained By the EFFECT Framework

We now further analyze features found by EFFECT on each of the three application settings. While ambiguous symbols were used in the alphabet for feature construction in the HS and ALU Site Detection problems, the basic set of {A, C, G, T} was used for the splice site recognition problem. During evaluation on 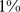 of training data, on which we tuned the methods employed for comparison to EFFECT in this paper, we found that symbol ambiguity was not useful for splice site recognition due to the presence of decoys.

#### HS Site Features

The entire HS dataset described above is used to obtain features through the EFFECT framework. Features reported are found to contain many compositional motifs, such as **CGCG**, **CGCGAGA**, and **(A/G)GG(T/G)**. Positional features with slight shifts recorded the presence of short 2-mers, such as **CG**, and long 8-mers, such as **CTTCCGCC**. Correlational features recorded the simultaneous presence of **GAT** and **ATCT**, and that of **CATTT** and **(G/T)GGC**. Interestingly, these last two features have been reported by other researchers as having important biological significance for maturation, silencer, and enhancer effects [118], [119]. Lastly, various features recorded the presence of **CG** patterns, such as **CGMS**, **CGMSN**, and **CGSBN**, which confirms current knowledge that HS sites are rich in **CG** nucleotides [25].

#### Splice Site Features

On the NN269 dataset, EFFECT reported many positional features, such as **(C/A)AGGTAAG** and **(T/C)(T/C)CCAGGT**. Note that these features match the donor and acceptor consensus sequences exactly. An interesting complex conjunction feature was reported, containing three positional features, **CG**, **GA**, and **AG**, around position 10 to 17 nt in the acceptor region. This is in good agreement with known acceptor region signals reported by other studies [120]. On the C_Elegans dataset, EFFECT reported many regional features, such as the 7-mer motifs **GGTAAGT**, **AGGTAAG**, and **GGTAGGT** around position -43 nt, matching the donor consensus sequence **AGGTAAGT**. Another important positional feature in the region -18 to-14 nt containing the **TAAT** motif was reported. We note that this motif s a well-known branch site signal [120]. Shift-Positional features around position -3 nt recorded the presence of motifs, such as **TTTCAGG** and **TTTCAGA**, matching the known acceptor consensus **TTTCAG(A/G)** sequence exactly.

#### ALU Site Features

**Figure 4:**
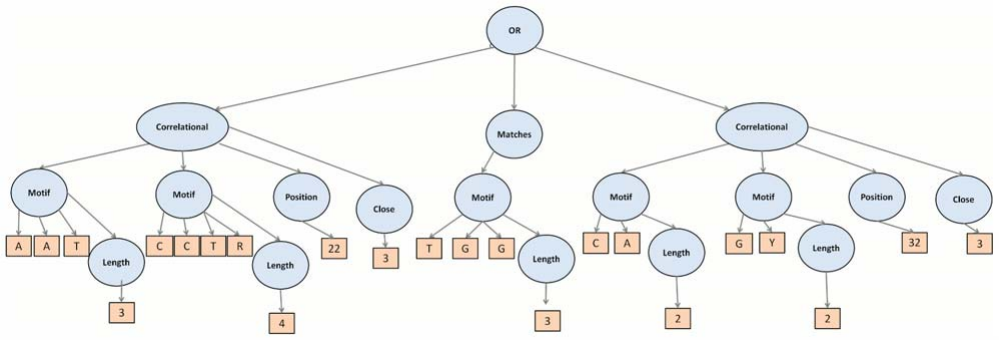
A complex disjunctive feature is obtained by the EFFECT framework for the ALU sequence classification problem. The feature is shown in the tree representation employed for features in this work.

**Figure 5:**
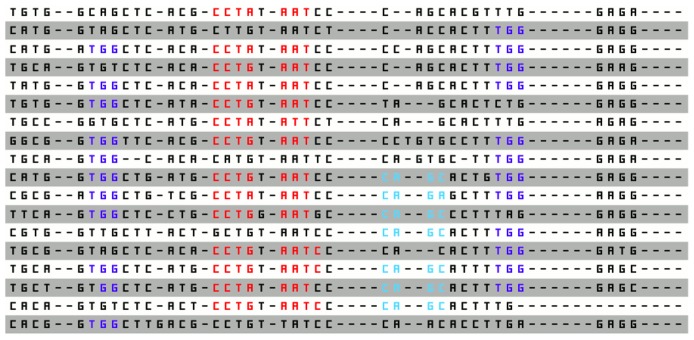
A clustal alignment of sequences shows the same overrepresented signals also combined as lower-level features in the disjunctive higher-level one found by the EFFECT framework.

On the ALU dataset, EFFECT reported many compositional features, such as motifs **AAAAAA**, **AAAAT**, **AGCCT**, **CCCAG**, and **CCTGT**. These are well known signals in ALU repeats [121]. An interesting disjunctive features was also reported, consisting of two correlational sub-features **CCTR, AAT, shift 3** and **CA, GY, shift 3** and a compositional feature **TGG**. This feature is shown in Figure 4. We additionally performed a clustal alignment on the whole ALU dataset, shown in Figure 5 and found the three sub-features of the disjunctive feature found by EFFECT and shown in Figure 4 to be indeed over-represented in the ALU dataset. This finding further highlights the importance of using ambiguous symbols in the representation for matching pyridines. Finally, additional disjunctive features recorded the presence of motifs, such as **CCTGG**, **CTGGGG**, and **GAGGC**, further showcasing the ability of EFFECT to combine the presence of lower-level signals in interesting higher-order features.

### Statistical Analysis of Obtained Features

Our detailed feature analysis concludes with measuring the information gain (IG) from each feature in the set reported by EFFECT. For a dataset D, with classes ranging from 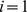 to k, the information theory metric for entropy, 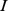, is given by:
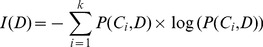
(4)


For a feature F taking on 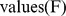 different values in 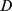, the weighted sum of its expected information (over splits of the dataset 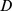 according to the different values of 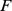 into 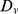 subsets, with 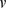 ranging from 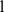 to 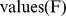) is given by:
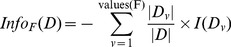
(5)


The information gain IG for a feature 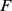 over a dataset 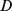 is then given by:
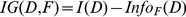
(6)


**Figure 6:**
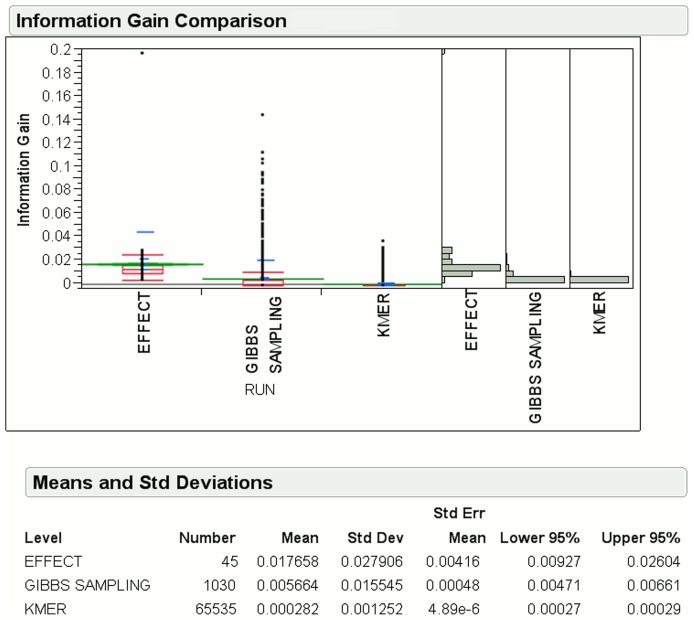
Information gain for features obtained by EFFECT on the HS site dataset is compared to that obtained by feature-based methods used for comparison in this work.

Figure 6 shows the mean information gain for EFFECT features is 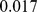, which is almost 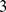 and 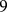 times more than that of the Gibbs sampling and k-mer methods, respectively, for HS sequences. We note that the number of features reported by EFFECT is 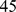, which is much smaller than the 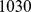 Gibbs sampling and 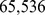 k-mer features.

**Figure 7:**
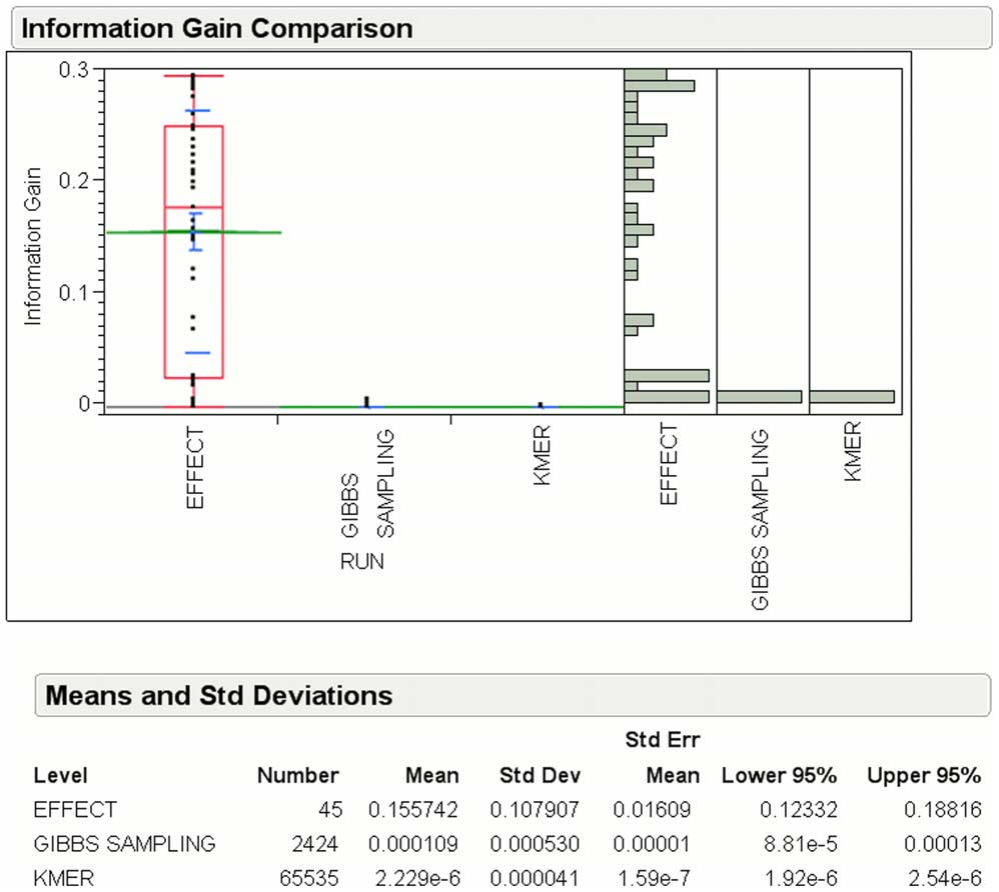
Information gain for features obtained by EFFECT on the NN269 Acceptor dataset is compared to that obtained by feature-based methods used for comparison in this work.

**Figure 8:**
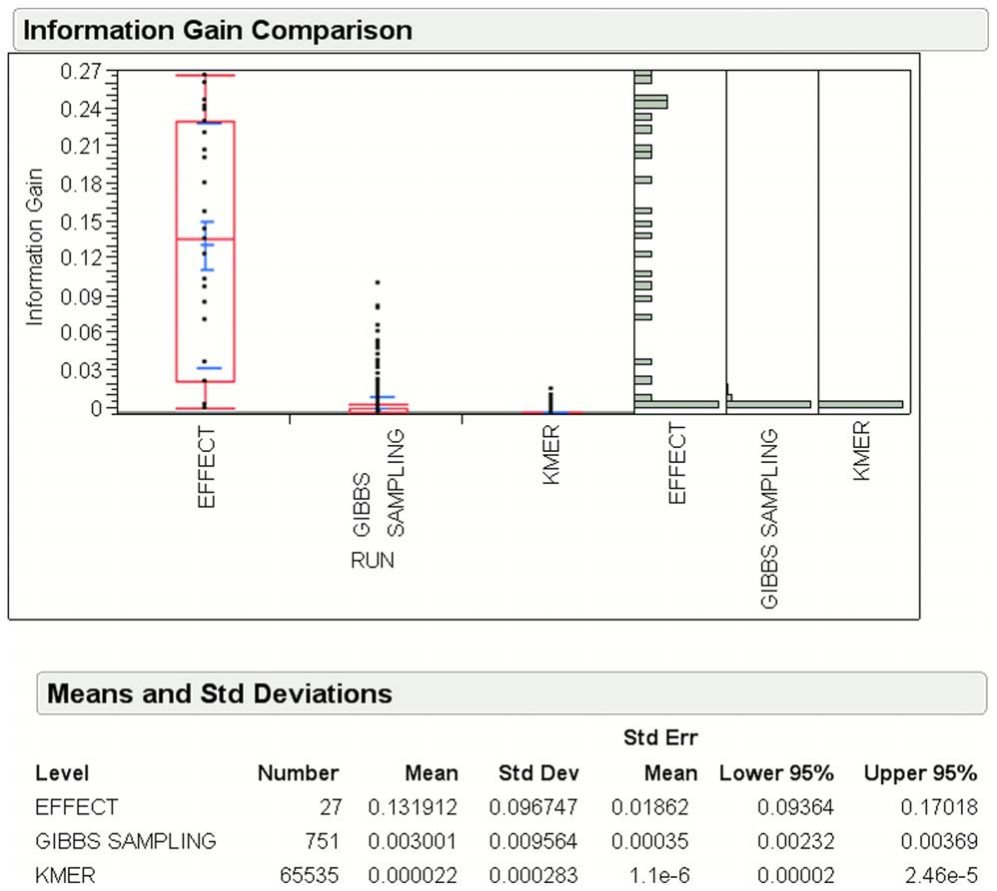
Information gain for features obtained by EFFECT on the NN269 Donor dataset is compared to that obtained by feature-based methods used for comparison in this work.

Figure 7 shows the mean information gain for EFFECT is 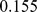, which is approximately 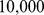 and 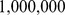 times more than that of the Gibbs sampling and k-mer methods, respectively, for acceptor splice sites. The number of features generated by EFFECT is only 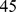, which is smaller than the 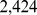 Gibbs sampling and 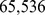 k-mer features. Figure 8 shows the mean information gain for EFFECT is 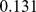, which is approximately 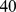 and 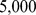 times more than that of the Gibbs sampling and K-mer methods, respectively, for donor splice sites. The number of features generated by EFFECT is only 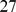, which is smaller than the 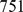 Gibbs sampling and 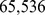 k-mer features.

**Figure 9:**
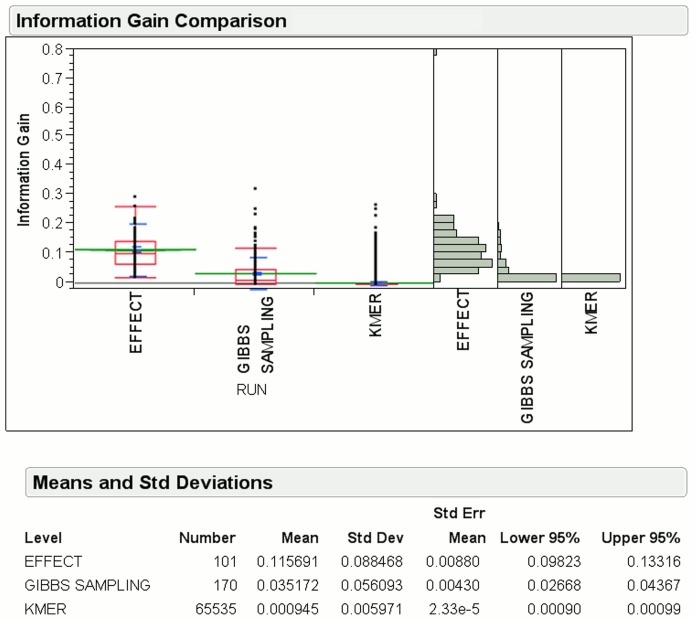
Information gain for features obtained by EFFECT on the ALU dataset is compared to that obtained by feature-based methods used for comparison in this work.

Figure 9 shows the mean information gain for EFFECT is 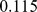, which is again approximately 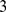 and 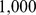 times more than that of the Gibbs sampling and k-mer methods, respectively, for ALU sequences. Also, the number of features generated by EFFECT is only 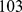, which is smaller than the 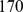 Gibbs sampling and 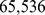 k-mer features.

Taken together, this analysis demonstrates that the EFFECT framework generates fewer but statistically more discriminating features, which is one of the most desired qualities required of feature construction algorithms.

## Discussion

In this paper we describe and evaluate EFFECT, a computational framework to automate the process of extracting discriminatory features for determining functional properties of biological sequences. Using basic domain knowledge to identify the fundamental building blocks of potential features, EFFECT constructs complex discriminatory features from these building blocks in a two-stage process. First, an evolutionary algorithm, EFC, constructs a set of potentially useful complex features. A second evolutionary algorithm, EFS, reduces the size of this feature set to a collectively effective subset.

The key to this approach is the use of a GP-based EA capable of efficiently constructing complex features from an appropriate set of basic building blocks. The generality of the approach is obtained by allowing more general building blocks than the basic sequence elements and by providing a flexible way of describing positional information. The effectiveness of the approach is enhanced by a novel feature selection phase. The power and the versatility of this approach is demonstrated by its application to three important problem areas: the recognition of hypersensitive, splice, and ALU sites in DNA sequences.

An important observation is the preciseness with which the constructed features characterize complex discriminatory patterns. Figure 5 illustrates some of the sequence patterns matched by the feature shown in Figure 4. If we imagine using basic spectrum K-mers for the same dataset, it would have taken a significant number of K-mers to capture the information. More importantly, the positional, correlational and compositional context would not have been captured. This would not only result in lower information gain at the cost of a higher number of features as clearly seen in the earlier analysis on information gain, but would also generate a large number false positives. Markov models and positional matrix-based algorithms would have captured more of the patterns outlined in the example, but not the complex combinations that EFC does.

In addition, the complex features constructed by EFFECT can frequently be interpreted in meaningful ways by the domain experts, providing additional insights into the determination of functional properties. Our web site describes the top constructed features obtained by EFFECT on the three application settings presented in this paper. We encourage interested researchers to study them directly for further insights.

Finally, we hope that the provided source code will provide the research community with a powerful tool to support further investigations in other application settings. For example, we note that interesting problems involving amino-acid sequences can be pursued with the EFFECT framework. In such settings, simple approaches involving enumeration of features is impractical, unless the amino-acid alphabet is drastically simplified. The proposed framework allows the exploring of large feature spaces while retaining more of the characteristics of amino acids. While further problem-specific details can be explored, the investigation can begin by simply replacing the DNA alphabet employed in this paper.

## Supporting Information

Figure S1 **Illustration of a compositional feature through the use of the matches operator.**
(TIF)

Figure S2 **Illustration of a positional feature through the use of the matchesAtPosition operator and Shift operators.**
(TIF)

Figure S3 **Illustration of a positional-shift feature through the use of the matchesAtPosition and Shift operators.**
(TIF)

Figure S4 **Illustration of a region-specific feature through the use of the matches and Region operators.**
(TIF)

Figure S5 **Illustration of a correlational feature that records the simultaneous presence of two features.**
(TIF)

Figure S6 **Illustration of the mutation operators in EFC.**
(TIF)

Figure S7 **Illustration of the crossover operator in EFC.**
(TIF)

Text S1 **Feature representation in EFC, population and generation mechanism in EFC, details on parameter tuning, statistical significance results, and comparison with other feature selection algorithms.** Table S1: Parameters used for experiments in HSS. Table S2: Parameters used for experiments in C Elegans (splice site). Table S3: Parameters used for experiments in Alu. Table S4: auROC and auPRC comparison analysis for HSS recognition. Table S5: auROC and auPRC comparison analysis for recognition of ALU sites. Table S6: auROC and auPRC comparison analysis with different feature selection methods. Table S7: Summary statistics are shown for information gain and number of features over 30 independent runs of EFFECT.
(PDF)
