# Supplementary figures and images for: Effective Automated Feature Construction and Selection for Classification of Biological Sequences
Source: PLoS One. 2014 Jul 17;9(7):e99982. doi: 10.1371/journal.pone.0099982 (PMC4102475; doi:10.1371/journal.pone.0099982)

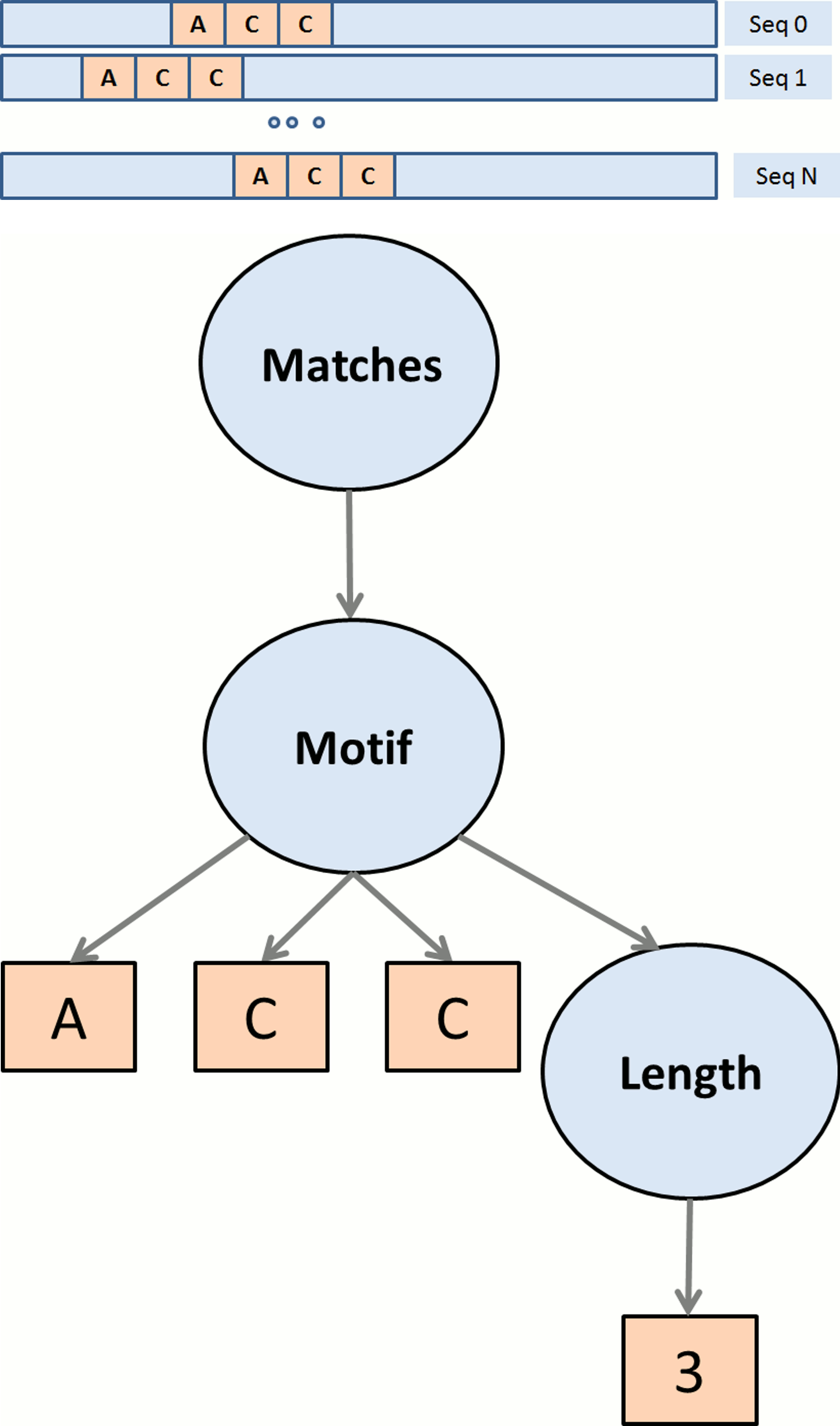

Supplement: Figure S1 — Illustration of a compositional feature through the use of the matches operator. (TIF) [file pone.0099982.s001.tif]

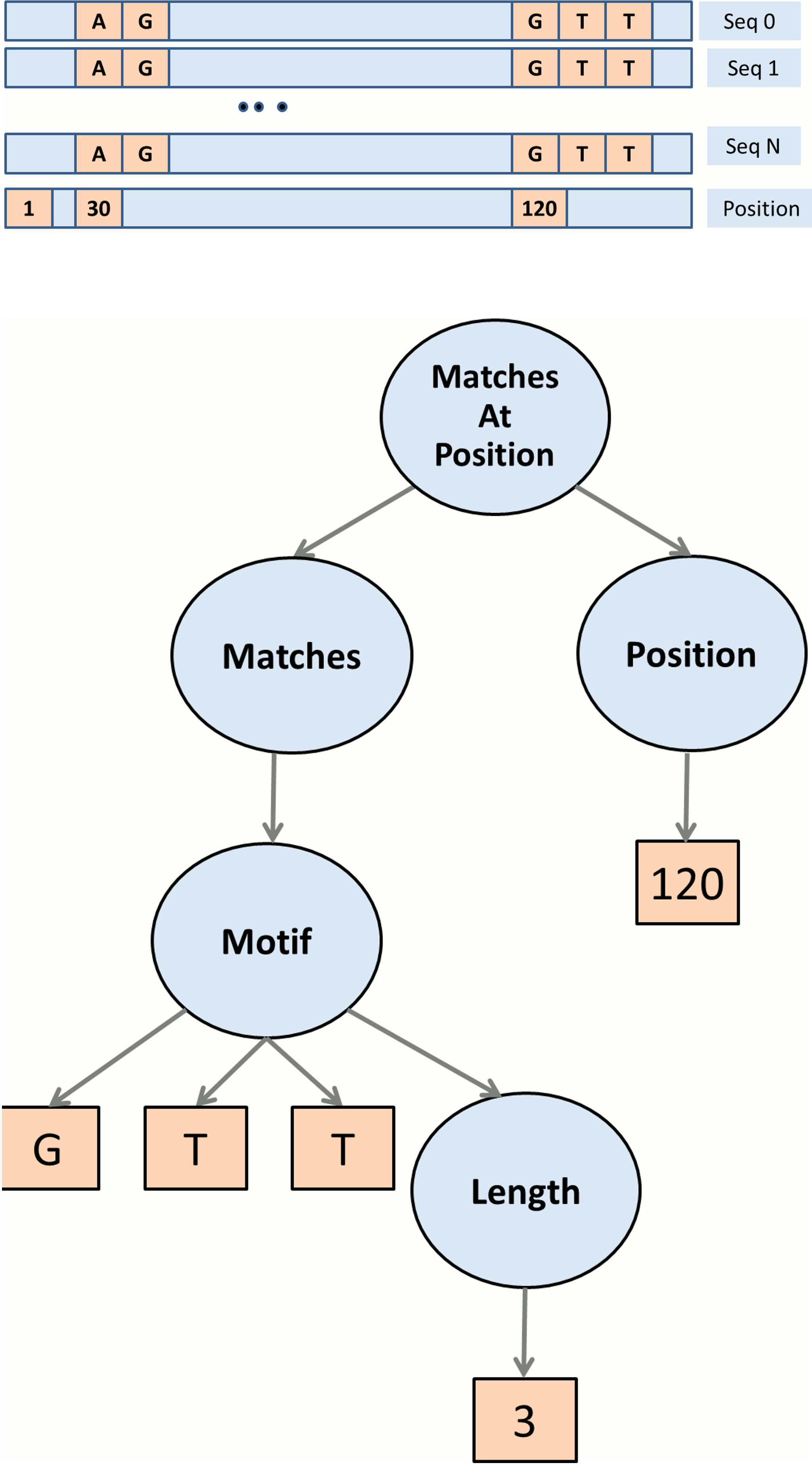

Supplement: Figure S2 — Illustration of a positional feature through the use of the matchesAtPosition operator and Shift operators. (TIF) [file pone.0099982.s002.tif]

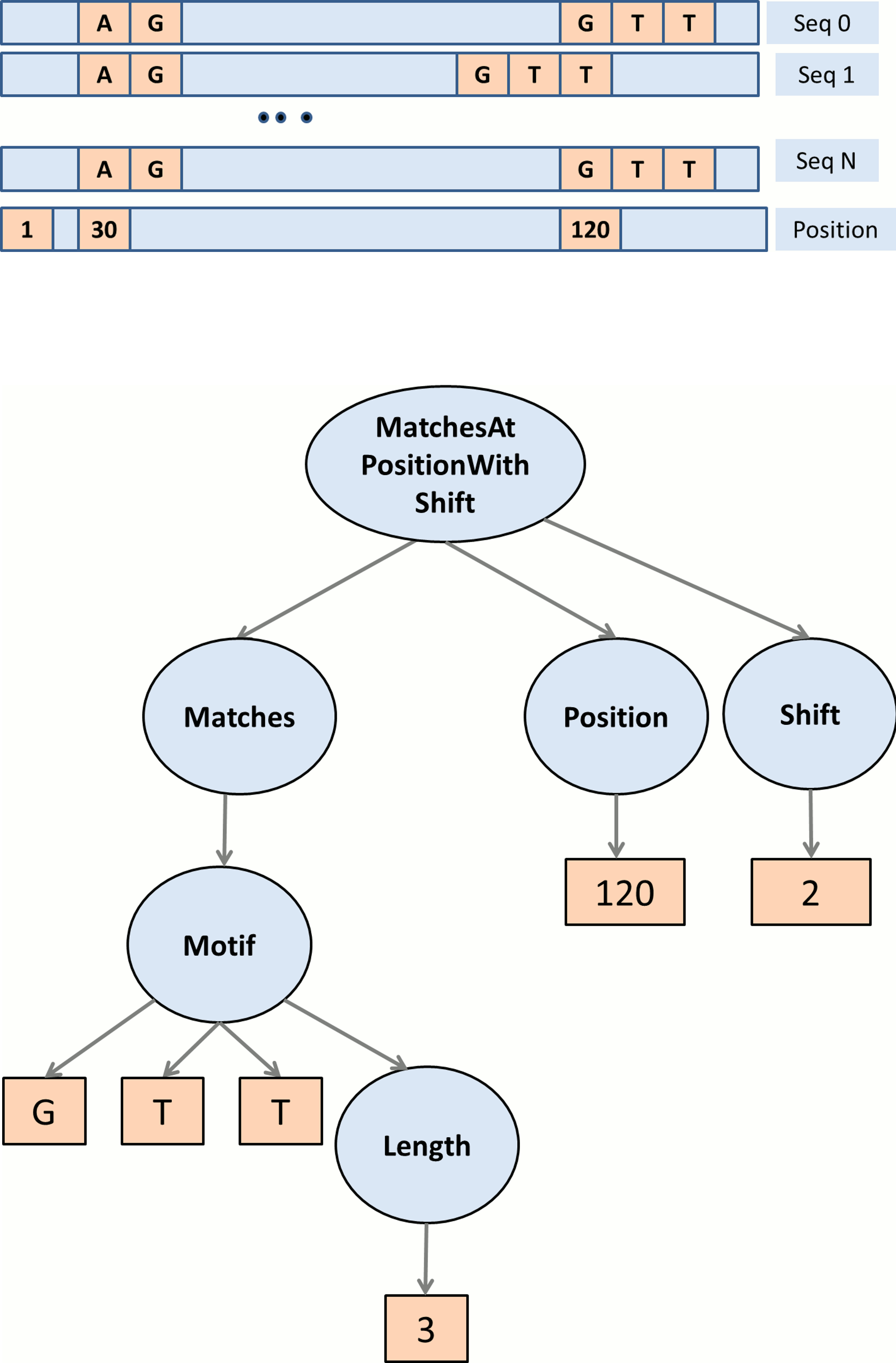

Supplement: Figure S3 — Illustration of a positional-shift feature through the use of the matchesAtPosition and Shift operators. (TIF) [file pone.0099982.s003.tif]

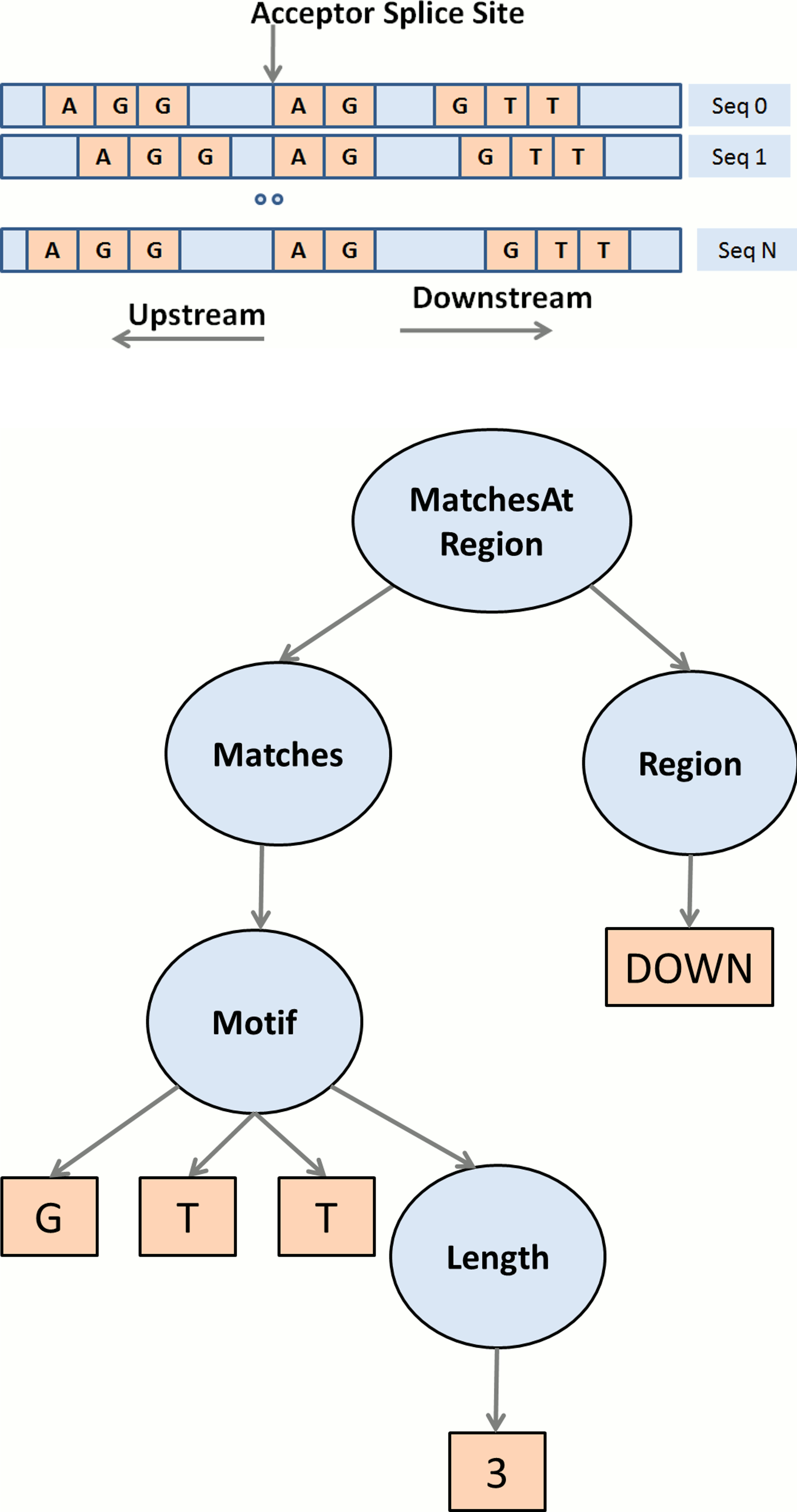

Supplement: Figure S4 — Illustration of a region-specific feature through the use of the matches and Region operators. (TIF) [file pone.0099982.s004.tif]

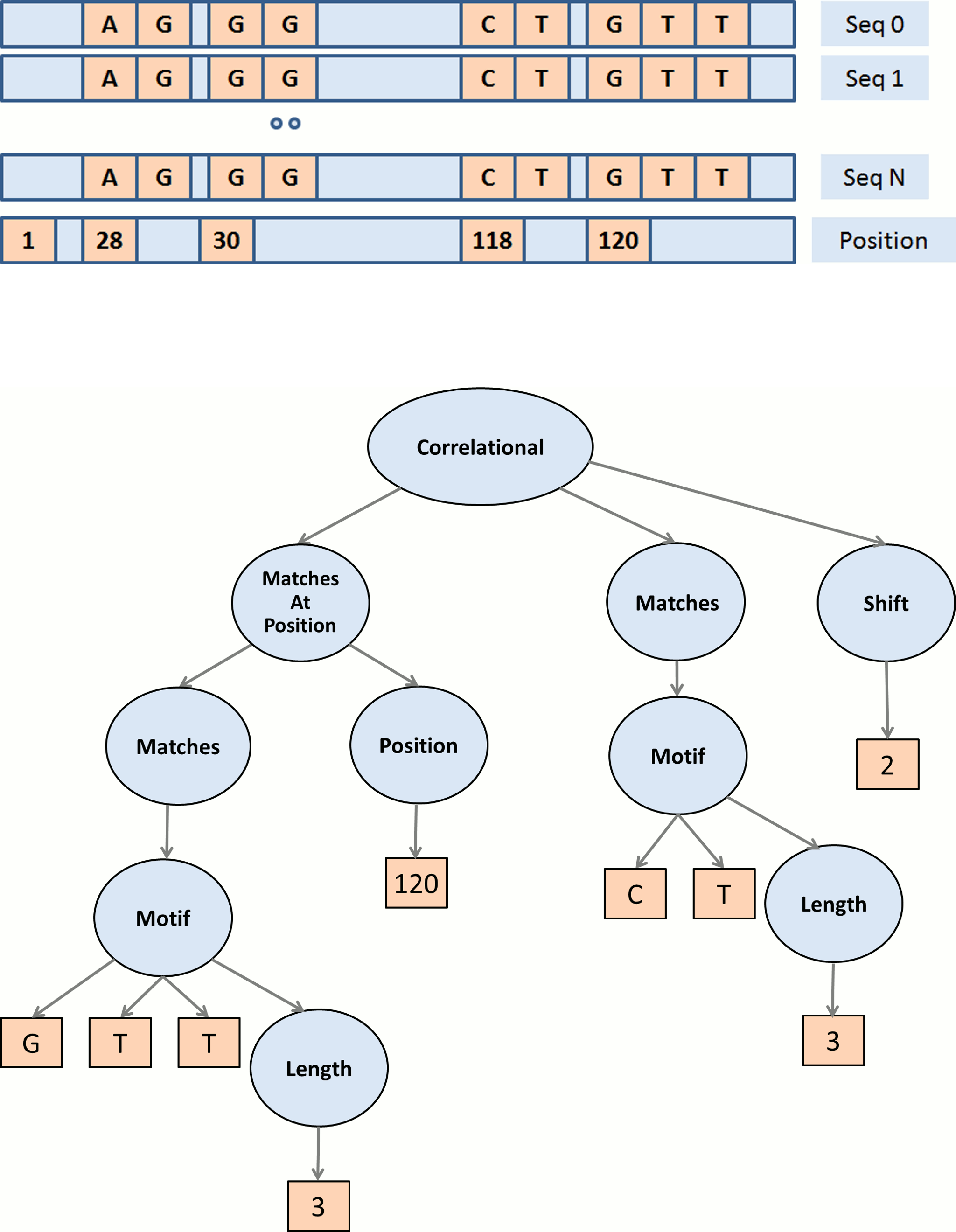

Supplement: Figure S5 — Illustration of a correlational feature that records the simultaneous presence of two features. (TIF) [file pone.0099982.s005.tif]
