# Supplementary material for: Effective Automated Feature Construction and Selection for Classification of Biological Sequences
Source: PLoS One. 2014 Jul 17;9(7):e99982. doi: 10.1371/journal.pone.0099982 (PMC4102475; doi:10.1371/journal.pone.0099982)
Supplement: Text S1 — Feature representation in EFC, population and generation mechanism in EFC, details on parameter tuning, statistical significance results, and comparison with other feature selection algorithms. Table S1: Parameters used for experiments in HSS. Table S2: Parameters used for experiments in C Elegans (splice site). Table S3: Parameters used for experiments in Alu. Table S4: auROC and auPRC comparison analysis for HSS recognition. Table S5: auROC and auPRC comparison analysis for recognition of ALU sites. Table S6: auROC and auPRC comparison analysis with different feature selection methods. Table S7: Summary statistics are shown for information gain and number of features over 30 independent runs of EFFECT. (PDF) [file pone.0099982.s008.pdf]

Supporting Information: Effective Automated Feature Construction and Selection for Classification of Biological Sequences

Uday Kamath[1], Kenneth De Jong[1,2,*], Amarda Shehu[1,3,4*]
1 Computer Science, 2 Krasnow Institute, 3 Bioengineering, 4 School of Systems Biology George Mason University , Fairfax, VA, USA
∗ E-mail: Corresponding authors [kdejong, amarda]@gmu.edu

1 Feature Representation in EFC

A key aspect of EFC's ability to construct relevant complex features is its use of functional primitives (operators) as building blocks.

Compositional Features The purpose of the `Matches` operator is to record the presence of a specific motif. Its arguments are a the symbols making up a particular motif and the length of the motif. As such, the `Matches` operator naturally allows encoding *global* compositional features. An illustration is provided in Supplementary Figure S1, where the showcased feature tree encodes the presence of the motif 'ACC' in a DNA sequence.

Positional Features In order to record the specific position in a sequence where a motif occurs and thus encode *local* positional features, a second operator, `MatchesAtPosition`, is employed. Its arguments are a compositional feature and a position. The compositional feature is encoded as above, through the use of the `Matches` operator. An illustration is provided in Supplementary Figure S2.

Positional-Shift Features The `MatchesAtPositionwithShift` operator allows constructing additional local positional features that may be displaced in either direction by a small shift. The shift can be provided as a parameter. Positional-shift features were discovered to be very effective in complex sequence/series classification problems such as splice site detection [1]. An example is provided in Supplementary Figure S3. The left subtree to the `MatchesAtPositionWithShift` operator is a `Positional` feature, and the right subtree is rooted at a `Shift` internal node.

Region-specific Features In some applications, the specific position is not important. Rather, recording the general location of a feature relative to a functional signal is more important. For instance, in splice site detection, it may be important to record whether a motif occurs downstream or upstream of the splice site. Region-specific features have been found to be important functional signals in sequence classification problems such as splice site detection [2,3]. Another operator, `Regional`, allows encoding such local features in EFC. As illustrated in Supplementary Figure S4, its arguments are a compositional feature in its left subtree and a right subtree rooted at `Region`.

Correlational Features Logically, there is no need for correlational features to be encoded explicitly, as they can be represented as sets of conjunctive features (one such conjunctive feature was illustrated above). However, in some applications, it may be computationally more effective to explicitly represent these features and simultaneously serve as a form of bloat control. EFC encourages this by providing an explicit `Correlational` operator node. An illustration of such a feature is shown in Supplementary Figure S5.

2 Population and Generation Mechanism in EFC

The initial population of features consists of N tree structures generated at random using the well-known *ramped half-and-half* method [4]. The method combines the *full* and *grow* techniques to provide a mixture of fully balanced and bushy GP trees. Half of the features in the initial population in EFC are obtained using the full technique, and the other half using the grow technique. A maximum depth of D is specified *a priori*, allowing the ramped half-and-half method to generate feature trees in the initial population with ramped depths in the range $\{1, \ldots, D\}$.

The full technique, which results in fully-balanced trees, recursively adds a non-terminal node to the tree (sampled at random over the list of non-terminals until the maximum depth D sampled uniformly at random in the $\{1, \ldots D\}$ range is reached. Terminal nodes are used at the leaf nodes. It is important to note that, as a GP algorithm, EFC relies on the principle of closure; that is, all generated trees are both syntactically and semantically correct. For example, once a non-terminal has been initialized, the sampling of the roots of its subtrees (including leaves) is limited to those that are correct arguments to the particular operator in the non-terminal. This constraint is also satisfied by the reproductive mechanisms that take one or two feature trees and modify them to obtain a new child feature that is syntactically and semantically correct.

The grow technique, which results in bushy trees, is similar to the full technique. However, the technique does not restrict the choice of nodes to non-terminals till maximum depth is reached. While the full technique results in fixed-shape trees, the grow technique results in trees of arbitrary shape. The purpose for using both the full and grow techniques is to obtain a diverse initial population, which is key to the ability of an EA to explore diverse regions in a potentially complex fitness landscape [4].

In our implementation of EFC in the EFFECT framework, we do not use the same fixed population size at each generation. Each subsequent generation reduces the size of the population by $r\%$ over the previous one. This strategy is known as *implosion* and is used to gradually apply selection pressure [5] and so address the aging problem observed in GP.

A population of features evolves for a pre-specified number of generations, set to 25 in EFC (analysis of fitness convergence shows this upper bound to be sufficient). Each population contributes its top ℓ features to a *hall of fame*. The purpose for employing a hall of fame is so that good features are not lost over generations but instead are preserved and serve as a global memory of the EFC. In turn, the hall of fame is used to initialize the next generation by contributing $m << N$ randomly selected features. sampled uniformly at random. Hall-of-fame mechanisms are widely used to improve performance when searching large spaces.

Reproductive Operators

Mutation The role of mutation is to make a small or local incremental change to a selected parent individual to form a new child individual. In GP, due to the representation of individuals through GP trees, the standard mutation operator is more disruptive than in other EAs. In the standard implementation, this operator samples an internal node at random and replaces the entire subtree rooted at the selected node with a new, randomly generated subtree [4]. In our implementation here, we pursue more controlled implementations that are problem-specific; in other words, our implementation of the mutation operator guarantees that the operator results in a small and incremental change. To achieve this, we introduce four mutation operators, a motif mutation, a positional mutation, a shift mutation, and an adjacency mutation, detailed below.

Supplementary Figure S7 illustrates how a selected parent individual subjected to some of these mutations results in a new individual as a child. Tournament selection over tournaments of size 7 is applied once to compare fitness values and select a winner as parent for mutation (the fitness function is detailed below). Once a parent is selected for mutation, any of the four mutation operators is sampled uniformly at random. If the domain of the operator is not found in the currently selected parent (for

instance, the position mutation is sampled, but the parent feature is a compositional one), a mutation operator is sampled anew, for a maximum of t trials.

Motif Mutation A motif mutation affects compositional and regional features. Any node with a motif return type in the tree is selected with probability P_n set to a small value of 0.1 in our implementation of EFC. A character in the motif is then selected equally at random and replaced with any letter of the alphabet. This process is similar to the standard bit-flip operation in EAs. If the alphabet character set has any mismatch characters, this form of change helps increase the range of string matching.

Position Mutation A position mutation affects positional positionWithShift, and correlational features. The value in the leaf of the subtree rooted at a Position internal node is changed to an integer sampled uniformly at random within the lengths of provided sequences. This allows exploring the presence of a particular motif at other sequence positions.

Shift Mutation A shift mutation affects positionWithShift features. The value in the leaf of the subtree rooted at a Shift internal node is changed to an integer sampled uniformly at random within the range specified for the shift.

Adjacency Mutation Finally, the adjacency mutation affects correlational features. This operator represents a larger change than the others above, as it replaces the entire left or right subtree of a correlational feature with a randomly sampled tree. The purpose of this mutation is to explore alternative adjacent motifs.

Crossover In this work, we employ a standard subtree crossover, which is one of the most common genetic recombination operators used in GP [4]. Subtree crossovers have been shown to allow GP form complex trees and thus explore vast search spaces more effectively. Unlike mutation, the magnitude of a change as the result of crossover can be very large, effectively placing a child feature anywhere in the feature space rather than a neighborhood of parent feature(s). Tournament selection with same tournament size of 7 is carried out twice to obtain two individuals from a population. Given two selected parent features, subtree crossover, illustrated in Supplementary Figure S8, finds a random node in each of the parent trees for swapping. If the selected nodes do not match in their return type, and swapping the subtrees rooted at these nodes does not violate the maximum depth constraint, then the swap is performed. Otherwise, the process is repeated for a maximum of 10 times to find another random position in the tree to swap. Successful swapping results in two child features, but only one is employed in EFC, effectively discarding the other resulting child feature.

Fitness Function

In EFC, the goal is to maximize the surrogate fitness function defined for features. However, traditional GP implementations are designed for fitness minimization [4]. So, in keeping with this, we define the Koza fitness of a feature f in EFC as $\text{Koza}(f) = 1/(\text{Fitness}(f))$. EFC then converts the Koza fitness back into the GP-adjusted fitness $1/(1 + \text{Koza}(f))$ to select fit individuals. Note that the GP-adjusted fitness takes values in $[0, 1]$.

Bloat Control

EFC algorithm uses two such strategies for bloat control. First, it encourages explicit representations of correlational features rather as a more complex set of conjunctive features. Second, it controls the

complexity of parents selected for mutation or crossover through lexicographic tournament selection: if multiple individuals have the same fitness, selection, the individual with the smaller tree depth is selected.

3 Parameter Tuning

We list here the tuned parameters for methods used in our comparative analysis. Those employed on the HS site recognition problem are listed in Supplementary Table S1, followed by those employed on the splice site recognition problem in Supplementary Table S2, and those employed on the Alu site recognition problem in Supplementary Table S3.

**Algorithms**	**Final Tuned Parameters**
K-mer	K=1 to 8
Gibbs Sampling	Motif Length Maximum =8
PWM-HMM	Sequence Length= Average(210), ess =4
BayesNetwork	Sequence Length= Average(210), ess =4,<br>Structure=InHomogenous Markov,95% confidence numerics<br>MixtureClassifier: algorithm=Quasi Newton Method,<br>method=CompositeLogPrior,<br>norm=true
HomogenousHMM	Sequence Length= Average(210), Markov Chain Order=4
WAM-HMM	Sequence Length= Average(210), Markov Chain Order=4
MSP	MixtureClassifier: algorithm= Quasi Newton Method,<br>epsilon stopping condition=1E-6,<br>epsilon line search =1E-4,<br>norm=true)<br>MSP=(PWM, HMM, CompositeLogPrior)
WeightedPosition	C=1.0, epsilon=1e-5, order=8, useSign=false
WeightedPositionWithShift	C=0.1, epsilon=1e-5, order=8, useSign=false,shift=3

Table S1. Parameters used for experiments in HSS.

Algorithms	Final Tuned Parameters
K-mer	K=1 to 8
Gibbs Sampling	Motif Length Maximum =8
PWM-HMM	Sequence Length=142, ess =4
BayesNetwork	Sequence Length=142, ess =4,<br>Structure=InHomogenous Markov,95% confidence numerics<br>MixtureClassifier: algorithm=Quasi Newton Method,<br>method=CompositeLogPrior,<br>norm=true
HomogenousHMM	Sequence Length=142, Markov Chain Order=2
WAM-HMM	Sequence Length= 142, Markov Chain Order=4
MSP	MixtureClassifier: algorithm= Quasi Newton Method,<br>epsilon stopping condition=1E-6,<br>epsilon line search =1E-3,<br>norm=true)<br>MSP=(PWM, HMM, CompositeLogPrior)
WeightedPosition	C=1.1, epsilon=1e-5, order=8, useSign=false
WeightedPositionWithShift	C=0.01, epsilon=1e-5, order=8, useSign=false,shift=3

Table S2. Parameters used for experiments in C_Elegans (splice site).

Algorithms	Final Tuned Parameters
K-mer	K=1 to 8
Gibbs Sampling	Motif Length Maximum =8
PWM-HMM	Sequence Length= Average(90), ess =4
BayesNetwork	Sequence Length= Average(90), ess =4,<br>Structure=InHomogenous Markov,95% confidence numerics<br>MixtureClassifier: algorithm=Quasi Newton Method,<br>method=CompositeLogPrior,<br>norm=true
HomogenousHMM	Sequence Length= Average(90), Markov Chain Order=4
WAM-HMM	Sequence Length= Average(90), Markov Chain Order=4
MSP	MixtureClassifier: algorithm= Quasi Newton Method,<br>epsilon stopping condition=1E-6,<br>epsilon line search =1E-4,<br>norm=true)<br>MSP=(PWM, HMM, CompositeLogPrior)
WeightedPosition	C=1.2, epsilon=1e-5, order=8, useSign=false
WeightedPositionWithShift	C=1.1, epsilon=1e-5, order=8, useSign=false,shift=3

Table S3. Parameters used for experiments in Alu.

4 Statistical Significance Results

In all experiments we employed Weka's paired-t tester implementation for measuring statistical significance. WeKa's implementation only outputs the algorithms which are significantly better, without explicitly listing p-values; mean and standard deviations are provided. Supplementary Table S4 shows means and standard deviations on the HSS recognition problem, and Supplementary Table S5 does so on the ALU recognition problem.

**Algorithm**	**auROC**	**auPRC**
Feature-based		
K-mer	82.20 (0.4)	82.6 (0.6)
Gibbs Sampling	79.3 (1.1)	50.3 (4.2)
EFFECT	**89.7 (0.4)**	**89.2 (0.5)**
Statistical-based		
PWM-HMM	70.8 (1.2)	47.8 (1.1)
BayesNetwork	72.5 (1.2)	49.5 (2.3)
HomogenousHMM	82.02 (0.78)	71.5 (0.89)
WAM-HMM	80.05 (0.9)	70.0 (0.67)
MSP	85.5 (0.34)	72.9 (0.23)
Kernel-based		
WeightedPosition	80.01 (0.22)	62.3 (0.9)
WeightedPositionShift	80.93 (0.25)	64.9 (1.1)

Table S4. auROC and auPRC comparison analysis for HSS recognition.

**Algorithm**	**auROC**
Feature	
K-mer	94.20 (0.2)
Gibbs Sampling	95.2 (0.35)
EFFECT	**98.9 (0.14)**
Statistical	
PWM-HMM	77.45 (1.3)
BayesNetwork	86.82 (1.6)
HomogenousHMM	93.6 (0.41)
WAM-HMM	94.59 (0.4)
MSP	93.54 (0.6)
Kernel	
WeightedPosition	96.9 (0.2)
WeightedPositionShift	97.8 (0.12)

Table S5. auROC and auPRC comparison analysis for recognition of ALU sites.

5 Feature Selection Algorithm Comparisons

We have conducted a comprehensive comparison of our EFS algorithm with a diverse list of other feature selection algorithms. Here we report this comparative analysis in the context of the HSS recognition problem. The methods used for comparison are state-of-the-art filter and wrapper-based methods. Since EFS narrows feature sets to around 50 features on average, we use the same as constraint while running the other algorithms. We use 10 folds cross-validation as described in the Methodology section in the manuscript for the purpose of this comparative analysis. Here is the list of feature selection methods employed for comparison, together with the search mechanism and parameters:

- mRMR (minimum Redundancy Maximum Relevance) [6]. Used with number of features=50 and selection= mutual information difference
- Correlation-based Feature Selection CFS (with Greedy Search) [7].
- FCBF (Fast Correlation based Search with SymmetricalUncertAttributeSet) [8]. Number of features=50.
- Relief-F (with Ranker) [9]. Number of features=50, neighbors=10 and sigma=2.
- Information Gain Ratio (with Ranking). Number of features=50.

Wrapper based feature selection methods

- Wrapper with SVM and with Greedy Search (WrapperSubsetGreedy). Linear SVM, 5 folds, thresold=0.01, C=1.0.
- Wrapper with SVM and with Genetic Algorithm Search (WrapperSubsetGenetic). Linear SVM, 5 folds, threshold=0.01, C=1.0, population=20, mutation=0.01, crossover=0.6, generations=20.

Supplementary Table S6 shows that there is no statistically-significant difference between EFS, WrapperSubSetGenetic and FCBF; however, EFS gives a better mean for both auROC and auPRC.

**Algorithm**	**auROC**	**auPRC**
Filter-based		
mRMR	71.49	52.6
CFS	84.7	78.5
FCBF	86.8	85.9
Relief-F	81.23	82.3
InfoGainRatio	73.3	63.3
EFFECT	89.7	89.2
Wrapper-based		
WrapperSubsetGreedy	81.8	81.5
WrapperSubsetGenetic	87.1	86.8

Table S6. auROC and auPRC comparison analysis with different feature selection methods.

6 Summary Statistics and other Analysis of Selected Features

Supplementary Table S7 shows some summary statistics on the information gain of features and number of features (selected by the feature selection algorithm) obtained over 30 independent runs of EFFECT.

The features obtained by EFFECT are not expected to be identical, given the stochastic nature of the feature generation and selection algorithms in the proposed framework. However, analysis of the 30 feature sets (obtained by running EFFECT 30 times) suggests that these sets share many features with one another. Due to the ambiguous alphabet and the presence of disjunctive features, answering the question of whether a feature appears in a feature set is not trivial. Nonetheless, we list here some interesting features that are found on the majority of the 30 runs on each of the three problems considered in this article. On the HSS recognition problem, a correlational feature capturing the presence of motifs 'GAT' and 'ATCT' was found in 30/30 of the halls of fame, and it was selected as a top feature in 27/30 runs. On the splice site recognition problem, conjunctional features over motifs 'AG', 'GA', and 'CG' were ranked as top features in 29/30 runs. This is not surprising, as these motifs are known biological signatures of splice sites. On the ALU recognition problem, motifs 'AAAAT' and 'TGG' were present as features by themselves or combined via disjunction and ranked as top features in 30/30 runs.

	**HSS**	**NN269 Acceptor**	**NN269 Donor**	**ALU**
InfoGain				
mean	0.02	0.158	0.141	0.12
std_dev	0.00012	0.05	0.03	0.001
max	0.04	0.19	0.188	0.131
min	0.013	0.09	0.07	0.101
Nr. of Features				
mean	43.1	47.1	24.3	108.1
std_dev	3.2	2.3	4.8	5.3

Table S7. Summary statistics are shown for information gain and number of features over 30 independent runs of EFFECT.

References

1. Sonnenburg S, Schweikert G, Philips P, Behr J, Rätsch G (2007) Accurate splice site prediction using support vector machines. BMC Bioinformatics 8: S7.

2. Islamaj-Dogan R, Getoor L, Wilbur WJ, Mount SM (2007) Features generated for computational splice-site prediction correspond to functional elements. BMC Bioinformatics 8: 410-416.

3. Pertea M, Lin X, Salzberg SL (2001) Genesplicer: a new computational method for splice site prediction. Nucl Acids Res 29: 1185-1190.

4. Koza JR (1992) On the Programming of Computers by Means of Natural Selection. Boston, MA: MIT Press.

5. Luke S, Balan GC, Panait L (2003) Population implosion in genetic programming. In: Genetic and Evolutionary Computation Conference. Springer-Verlag, volume 2724, pp. 1729-1739.

6. Peng H, Long F, Ding C (2005) Feature selection based on mutual information: criteria of max-dependency, max-relevance, and min-redundancy. IEEE Transactions on Pattern Analysis and Machine Intelligence 27: 1226–1238.

7. Hall MA (1999) Correlation-based Feature Selection for Machine Learning. Ph.D. thesis, University of Waikato, Hamilton, New Zealand.

8. Song Q, Ni J, Wang G (2013) A fast clustering-based feature subset selection algorithm for high-dimensional data. IEEE Transactions on Knowledge and Data Engineering 25: 1-14.

9. Liu H, Motoda H, editors (2008) Computational Methods of Feature Selection. Chapman & Hall.

Figure Legends

Figure S1

Illustration of a compositional feature through the use of the `matches` operator.

Figure S2

Illustration of a positional feature through the use of the `matchesAtPosition` operator and `Shift` operators.

Figure S3

Illustration of a positional-shift feature through the use of the `matchesAtPosition` and `Shift` operators.

Figure S4

Illustration of a region-specific feature through the use of the `matches` and `Region` operators.

Figure S5

Illustration of a correlational feature that records the simultaneous presence of two features.

Figure S7

Illustration of the mutation operators in EFC.

Figure S8

Illustration of the crossover operator in EFC.

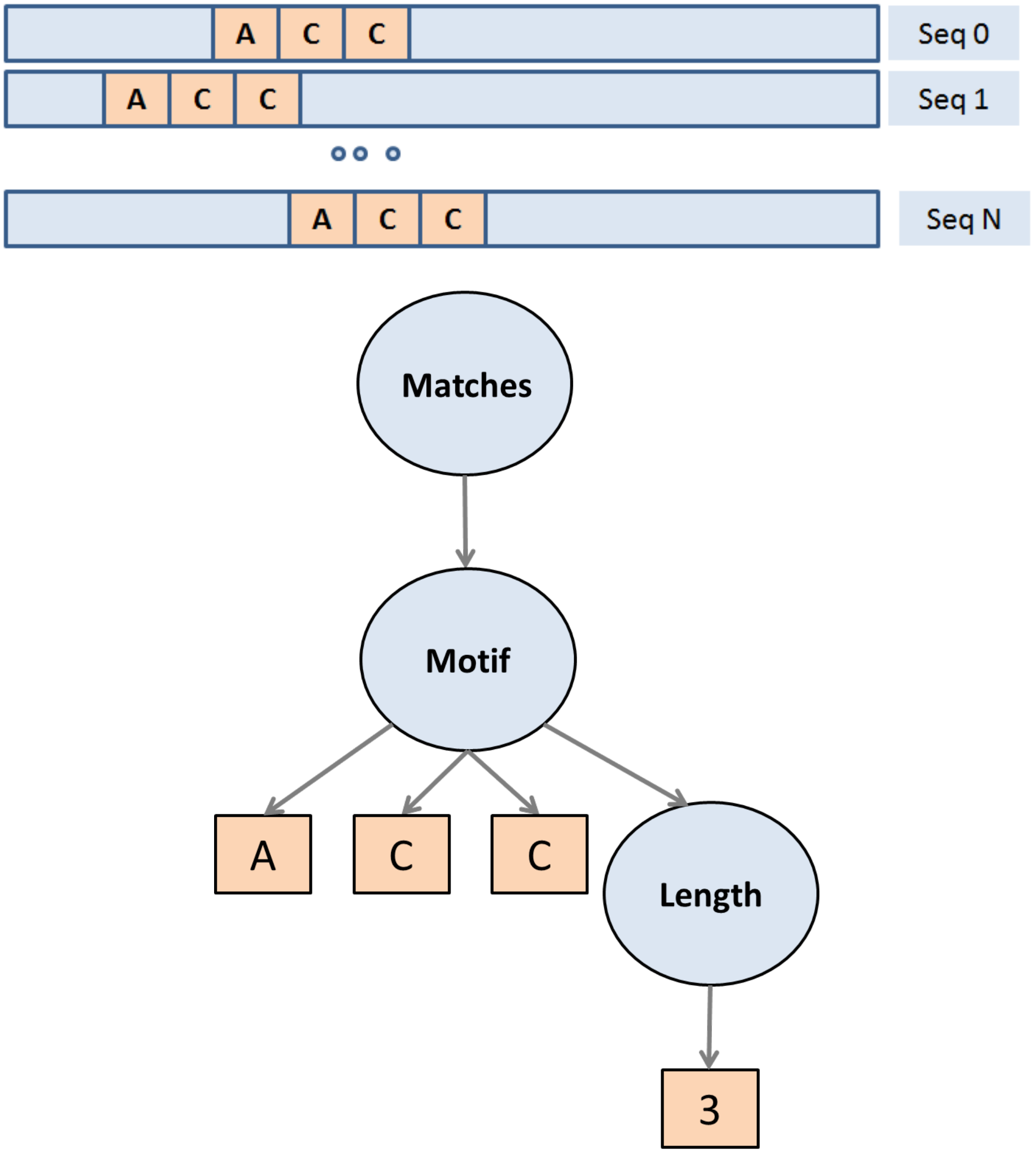

Figure S1

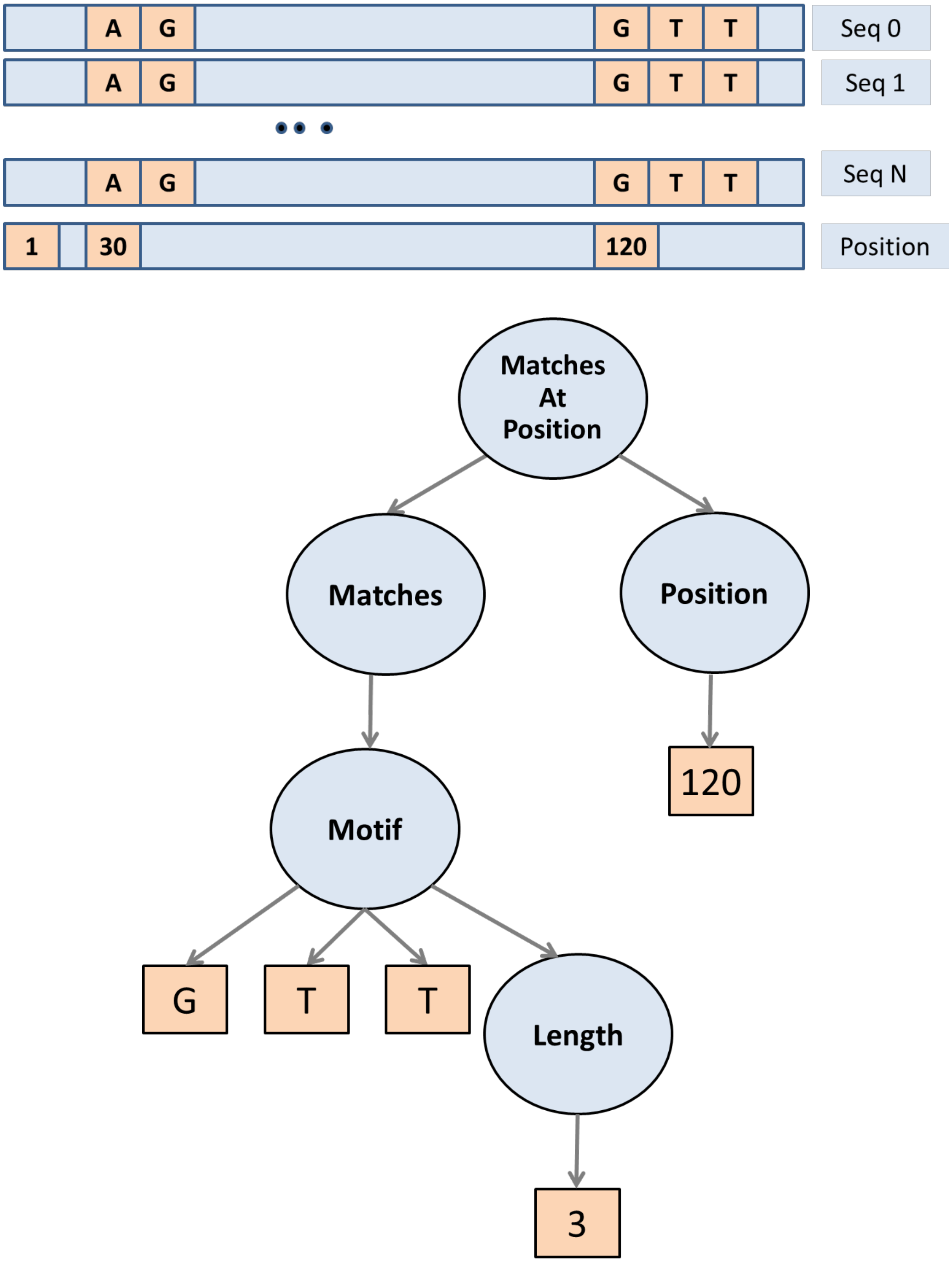
A
G
G
T
T
Seq 0
Seq 1
Seq N
1
30
120
Position
Matches
At
Position
Matches
Position
120
Motif
G
T
T
Length
3

Figure S2

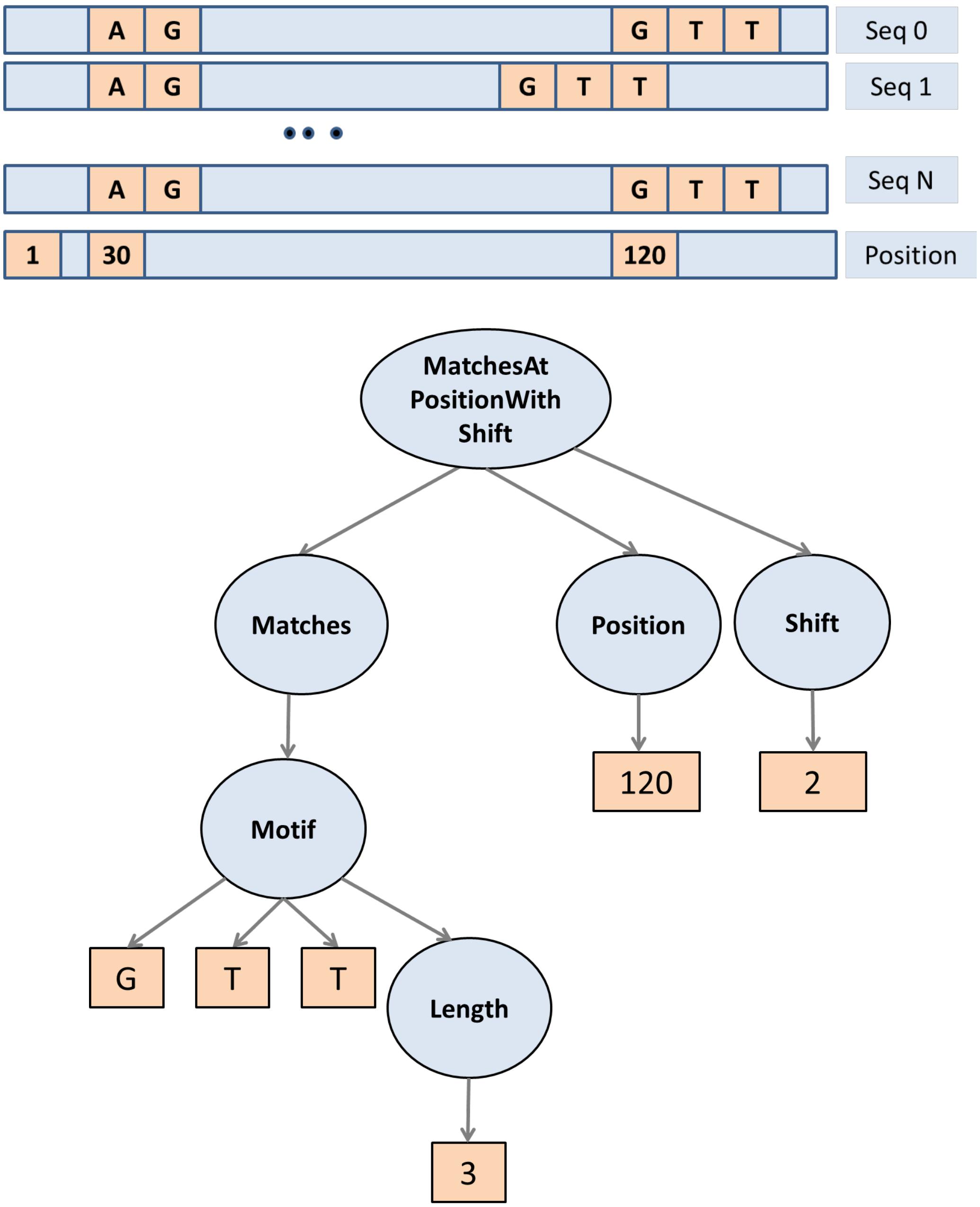
A
G
G
T
T
Seq 0
A
G
G
T
T
Seq 1
A
G
G
T
T
Seq N
1
30
120
Position
MatchesAt PositionWith Shift
Matches
Position
Shift
120
2
Motif
G
T
T
Length
3

Figure S3

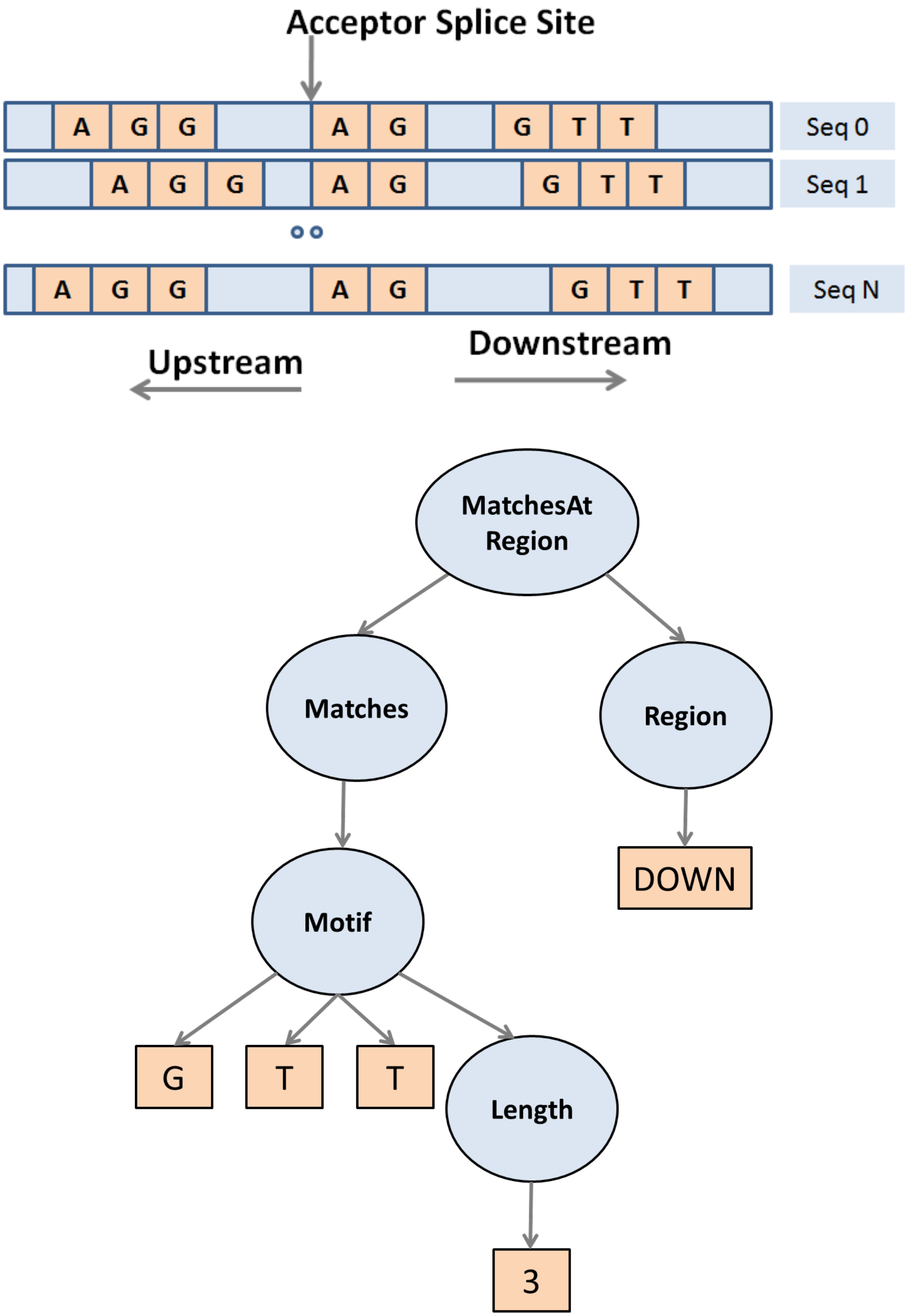
Acceptor Splice Site
A G G A G G T T
Seq 0
A G G A G G T T
Seq 1
A G G A G G T T
Seq N
Upstream
Downstream
MatchesAt Region
Matches
Region
Motif
DOWN
G
T
T
Length
3

Figure S4

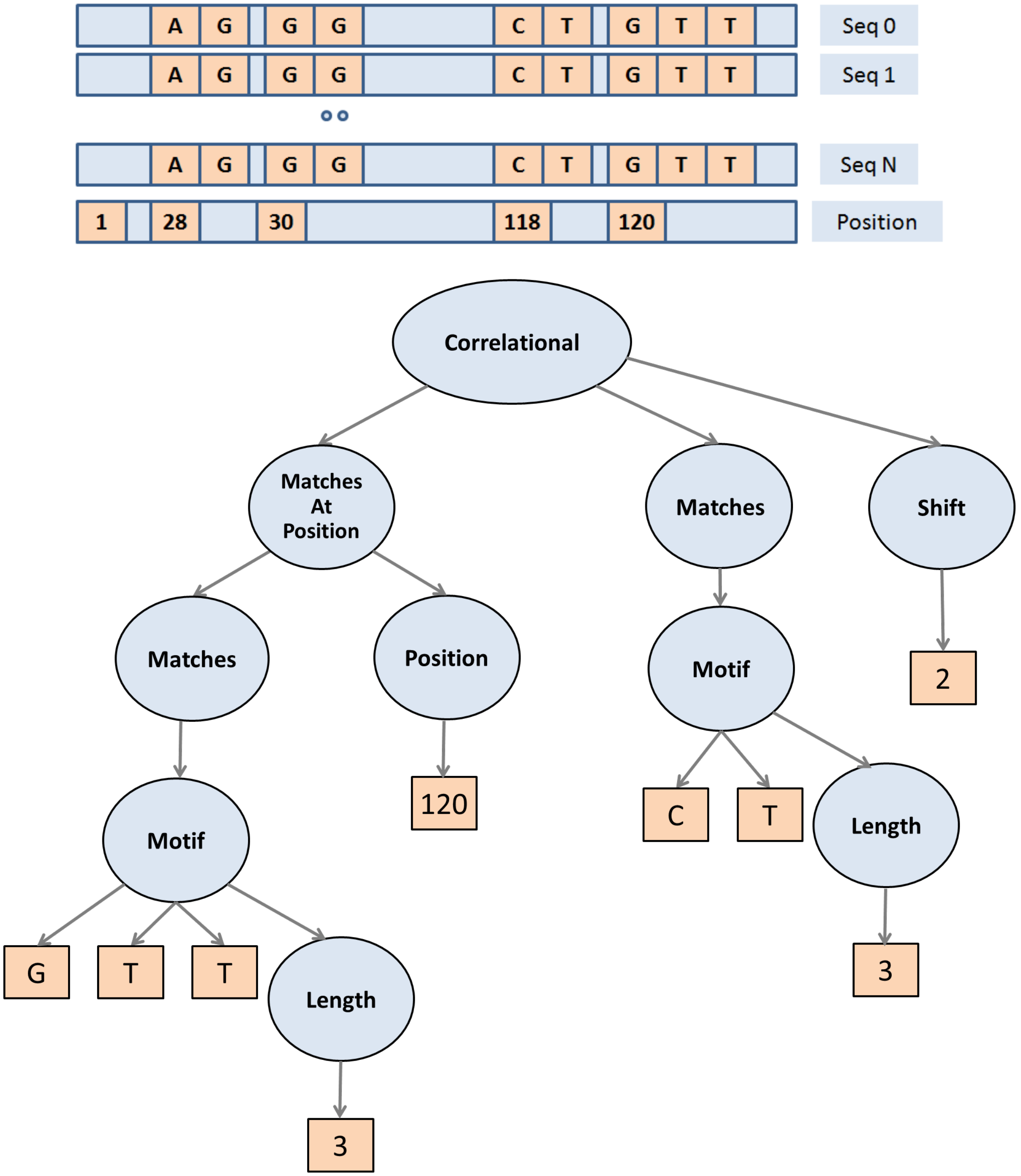

A G G G C T G T T Seq 0
A G G G C T G T T Seq 1
A G G G C T G T T Seq N
1 28 30 118 120 Position
Correlational
Matches At Position
Matches
Shift
Matches
Position
Motif
2
Motif
120
C
T
Length
G
T
T
Length
3
3

Figure S5

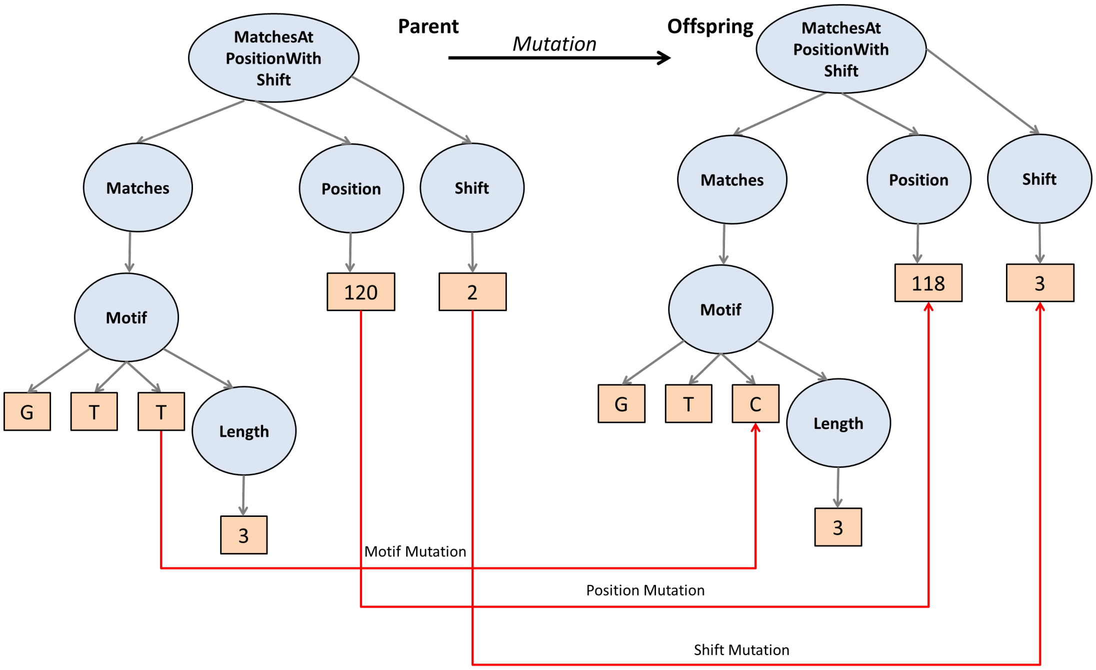

Figure S6

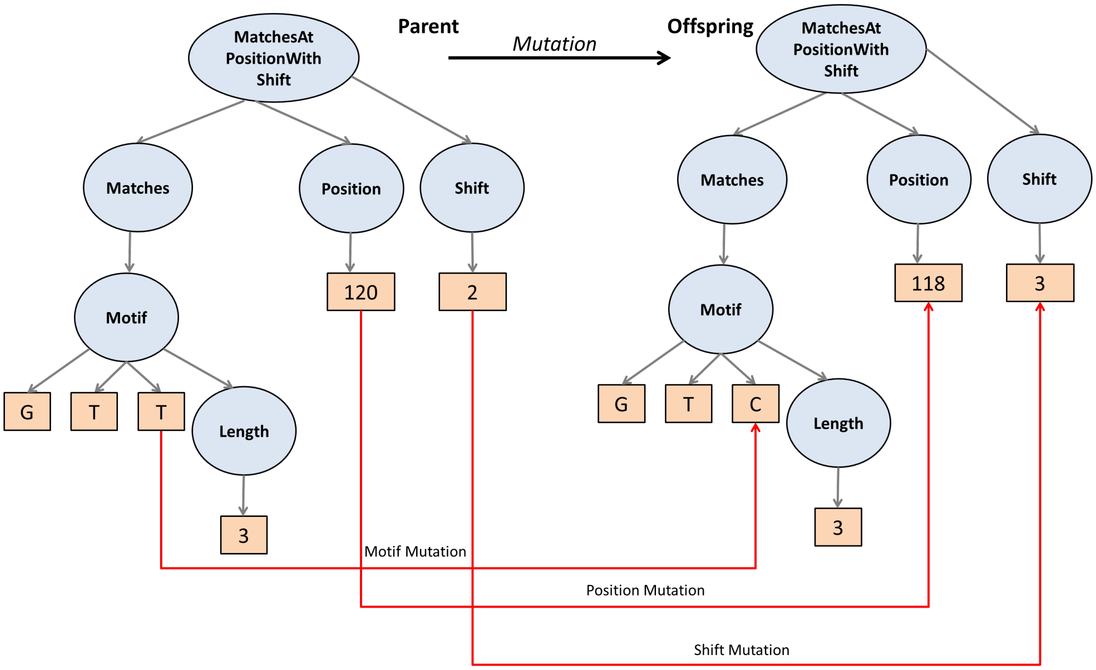

Figure S7

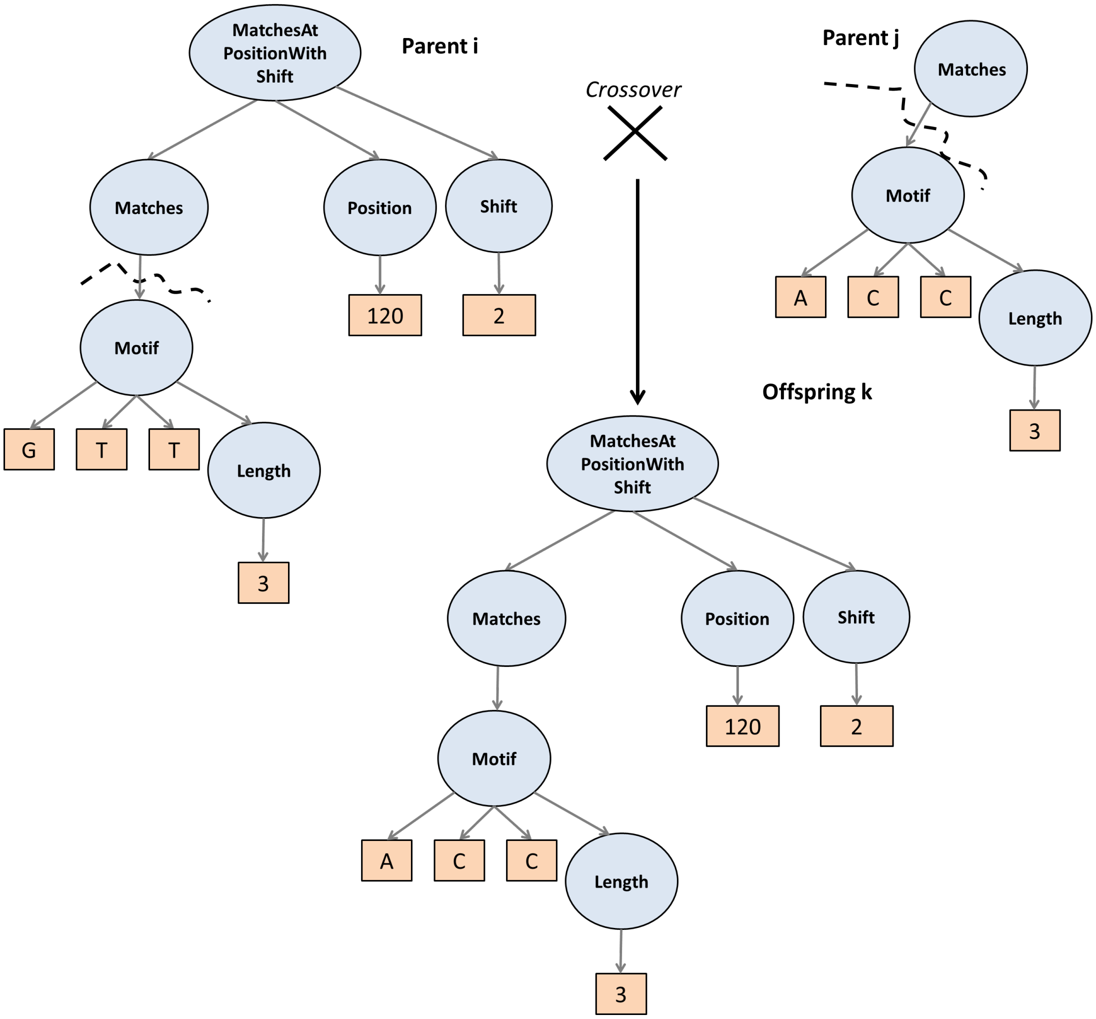
MatchesAt
PositionWith
Shift
Parent i
Matches
Position
Shift
120
2
Motif
G
T
T
Length
3
Crossover
Parent j
Matches
Motif
A
C
C
Length
3
Offspring k
MatchesAt
PositionWith
Shift
Matches
Position
Shift
120
2
Motif
A
C
C
Length
3

Figure S8
